# Identification of MYH9 Key Domain Involved in the Entry of PRRSV Into Permissive Cells

**DOI:** 10.3389/fmicb.2022.865343

**Published:** 2022-05-25

**Authors:** Liangliang Li, Weiyao Sun, Qifan Hu, Tongtong Wang, Guang Zhu, Qin Zhao, En-Min Zhou

**Affiliations:** ^1^College of Agronomy, Liaocheng University, Liaocheng, China; ^2^Department of Preventive Veterinary Medicine, College of Veterinary Medicine, Northwest A and F University, Xianyang, China; ^3^Shandong Vocational Animal Science and Veterinary College, Weifang, China

**Keywords:** PRRSV, MYH9, virus entry, aa1676-1791, antiviral

## Abstract

Porcine reproductive and respiratory syndrome virus (PRRSV) is an important pathogen that causes huge losses economically to the pig industry worldwide. Previous research suggested that receptor dependence is necessary for PRRSV infection. MYH9 and CD163 are indispensable for PRRSV entry into a porcine alveolar macrophage. In the present study, human MYH9 (hMYH9) and mouse MYH9 (mMYH9), similar to swine MYH9, could also accelerate PRRSV infection in pCD163-mediated cell lines. Knockdown of MYH9 activity using the specific small interfering RNA or inhibitor (blebbistatin) concomitantly decreased PRRSV infection. C-terminal fragment of MYH9 (PRA) proteins from different mammalian species contains a conserved binding domain (aa1676-1791) for PRRSV binding, since the recombinant MYH9^1676−1791^protein could inhibit the PRRSV infection significantly. Furthermore, the specific polyclonal antibody of MYH9^1676−1791^ could block PRRSV infection in host cells. These data strongly supported that MYH9, a very important cofactor, participated in PRRSV entry into target cells, which may facilitate the development of a new therapeutic agent to control PRRSV infection.

## Introduction

The porcine reproductive and respiratory syndrome virus (PRRSV) causes an economically important disease worldwide to the swine industry. Until now, PRRSV is known to specifically infect swine, and no available data show that other species are susceptible to this etiology. PRRSV has a narrow cell tropism for cells both *in vivo* and *in vitro* (Duan et al., 1998; Teifke et al., 2001). To date, host factors involved in the PRRSV cellular tropism are still not completely understood, and several entry mediators have been identified for PRRSV, which include heparin sulfate (HS) (Delputte et al., 2002), vimentin (Kim et al., 2006), CD151 (Wu et al., 2014), porcine CD163 (pCD163) (Van Gorp et al., 2010), sialoadhesin (CD169), DC-SIGN (CD209) (Pineyro et al., 2016), and MYH9 (Gao et al., 2016; Guo et al., 2016). Among them, MYH9 and pCD163 have been proved to be indispensable for PRRSV infection (Burkard et al., 2017; Ma et al., 2017; Wells et al., 2017; Hou et al., 2019).

Our knowledge about MYH9 has been rapidly updated in recent years. MYH9 is a cellular motor protein that is involved in cell–cell adhesion, integrin-mediated adhesion, epithelial cell polarization, cell migration, and morphogenesis (Vicente-Manzanares et al., 2009). Meanwhile, another function of MYH9 is that it acts as a cell receptor or factor that participates in the internalization and redistribution of several viruses on the plasma membrane, such as herpes simplex virus-1 (HSV-1), severe fever with thrombocytopenia syndrome virus (SFTSV), Epstein-Barr virus (EBV), PRRSV, and SARS-COV-2 (Arii et al., 2010; Sun et al., 2014; Xiong et al., 2015; Chen et al., 2021). For PRRSV infection, MYH9 served as an important cofactor for establishing the PRRSV infection. MYH9 can not only redistribute itself on the cell membrane and bind to PRRSV GP5 protein during viral internalization stages (Gao et al., 2016; Li et al., 2019; Xue et al., 2019), but can also participate in the cell-to-cell spread of PRRSV via nanotubes (Guo et al., 2016).

During the entry of PRRSV into the susceptible host cell, pCD163 is the key receptor and interacts with the minor envelope glycoproteins GP2a and GP4 of PRRSV, where the GP2a and GP4 proteins serve as the viral attachment proteins (Das et al., 2010, 2011). A previous study showed that the CD163 cDNAs derived from pig, dog, mouse, human, and African green monkeys could render non-permissive cell lines of PRRSV (e.g., BHK-21, PK032495, NLFK, and LLC-PK cells) to become permissive for PRRSV (Calvert et al., 2007), which indicated the CD163 homologs from other mammalian species could serve as PRRSV receptor. However, the information of MYH9 homologs from other mammals (human and mouse) mediated PRRSV entry is limit.

In the present study, we first determined whether MYH9 homologs from other mammalian species also showed PRRSV entry mediator activity. Furthermore, the key domain of MYH9 for PRRSV entry was identified at aa 1676–1791. Meanwhile, the anti- MYH9^1676−1791^ serum could reduce PRRSV infection. Our study not only complements the knowledge on MYH9 during PRRSV infection but is also helpful for the development of an antiviral peptide to prevent PRRSV infection in pigs.

## Materials and Methods

### Cells, Viruses, and Antibody

Porcine alveolar macrophage cells (PAMs) were collected from PRRSV-negative pigs (6-week-old) as previously reported (Li et al., 2017) and maintained in RPMI-1640 medium supplemented with 10% fetal bovine serum (FBS). MARC-145 cells were purchased from China Center for Type Culture Collection (CCTCC, Wuhan, China). All cells were cultured in Dulbecco's minimal essential medium (DMEM) with 10% FBS (FBS, Gibco, Carlsbad, CA, USA). PK-15^CD163^ cell lines were established and stored in our lab (Wang et al., 2013). The following isolates of PRRSV were selected for the cultures: PRRSV-2 isolates SD16 (GenBank: JX087437.1), CH-1a (GenBank: AY032626), JXA1 (GenBank: EF112445.1), GD-HD (GenBank: KP793736.1), and VR-2332 (GenBank: EF536003.1), and PRRSV-1 isolates GZ11-G1 (GenBank: KF001144.1) and P073-3 (only partially sequenced and confirmed as a PRRSV-1 isolate, and full sequence is unavailable). Monoclonal mouse antibodies against N protein of PRRSV (6D10), Mab2-5G2, and anti-pCD163 polyclonal antibodies were produced in our lab. Mouse anti-β-actin antibody, 4,6-diamidino−2-phenylindole (DAPI), and blebbistatin (a selective myosin II inhibitor) were purchased from Sigma-Aldrich (St. Louis, MO, USA). HRP-conjugated goat anti-mouse IgG antibodies and Texas Red-affiniPure goat anti-mouse IgG (H+L) were purchased from Jackson ImmunoReseach (West Grove, PA).

### Establishment of Recombinant Cell Lines Expressing pCD163 and PRRSV Infection

The porcine CD163 gene (GenBank ID: JX292263) was amplified and cloned into a pTRIP-CMV-Puro vector. Lentivirus production was performed as previously described (Du and Tikoo, 2010; Li et al., 2017). HEK-293T and BHK-21 cells were transduced with lentivirus pTRIP-CMV-CD163-IRES-puro and screened with puromycin according to the previously reported protocol (Gao et al., 2014; Li et al., 2017), and the new cell lines were termed as HEK-293T^CD163^ and BHK-21^CD163^, respectively. The susceptibility of HEK-293T^CD163^ and BHK-21^CD163^ cell lines to PRRSV was determined, and the cell lines thus obtained were referred to as PK-15^CD163^ (Wang et al., 2013). Briefly, these cells were inoculated with PRRSV SD16 strain at 1 MOI, and then the cells and cellular supernatant were collected at 48 h post-infection (hpi). The PRRSV replication was determined by Western blot and TCID_50_, and these cells were also assessed for their susceptibility to other strains of PRRSV (SD16, VR-2332, JXA1, and GD-HD) at 1 MOI using indirect immunofluorescent assay (IFA) at 48 hpi.

### Generation of the Recombinant PRA From Other Species and Truncated PRA

Total RNA was extracted from HEK-293T, BHK-21, and MARC-145 cells using TRIzol reagent (Thermo Fisher, USA), respectively. The C-terminal domain of MYH9 (aa 1651–1960) was designated as PRA, and the PRA fragments from other species (human and mouse) and truncated PRA were amplified (primers are listed in Table 1) and cloned into pCold-SUMO vector with the same enzymes *Sal*I and *Xba*I (Haigene, China). All sequences were confirmed by sequencing analysis (Sangon Biotech, China). These recombinant expression plasmids were transformed into the *E.coli* strain BL21 (DE3), respectively, and the different PRA proteins were expressed and purified according to a previous study (Li et al., 2018).

**Table 1:** The sequences of siRNAs and Primers for qPCR used in this study.

**Genes name**	**Sequence (sense 5** **′** **-3** **′)**	**Sequence (anti-sense 5** **′** **-3** **′)**
siRNA-1136	GCAUCAACGUGACCGAUUUTT	AAAUCGGUCACGUUGAUGCTT
siRNA-2245	CGUGCGUGCUCAUGAUAAATT	UUUAUCAUGAGCACGCACGTT
siRNA-913	GGUGCCAACAUUGAGACUUTT	AAGUCUCAAUGUUGGCACCTT
siRNA-NC	UUCUCCGAACGUGUCACGUTT	ACGUGACACGUUCGGAGAATT
GAPDH	CCTTCCGTGTCCCTACTGCCAAC	GACGCCTGCTTCACCACCTTCT
ORF-7^a^	ATGGCCGGTAAAAATCAGAGCC	TTAATTCGCACCCTGACTGG
ORF-7^b^	ATGCCAAATAACAACGGCAAGCAGC	TCATGCTGAGGGTGATGCTGTG

*^a^PRRSV-1.*

*^b^PRRSV-2*.

### Inhibition of Virus Infection Assays

PK-15^CD163^, BHK-21^CD163^, and HEK-293T^CD163^ cells were cultured and pretreated with blebbistatin inhibitor at the indicated concentrations for 30 min, and then inoculated with PRRSV SD16 or VR-2332 strain at an MOI of 1, respectively. After removing the medium, the cells were cultured with a 3% FBS DMEM medium containing the same concentrations of blebbistatin. The N protein in mRNA and protein levels of PK-15^CD163^, BHK-21^CD163^, and HEK-293T^CD163^ cells were analyzed by IFA at 36 hpi.

The PRA proteins from other species (human and mouse) and truncated PRA were diluted serially and incubated with PRRSV SD16 at 0.1 MOI. The mixture was maintained at 37°C for 1 h, then PAM cells were added to 6-well plates at 37°C and incubated for 1 h, and later washed with PBS to remove the unbound virus. The mRNA level of the PRRSV-2 type nucleocapsid (N) gene and expression level of protein were evaluated via qPCR and Western blot at 24 hpi. The mRNA level of PRRSV-1 type nucleocapsid (N) gene and the copies of the RNA genome were tested by qPCR according to the protocol described in a previous study (Li et al., 2018).

For antibody blocking assays, PAM and MARC-145 cells were pretreated with anti-MYH9^1676−1791^ polyclonal antibody produced in our lab, which was serially diluted to indicated concentrations in RPMI-1640 or DMEM, or with control mouse serum for 1 h at 37°C. Then, the cells were infected with PRRSV without removing the antibodies. The infected cells were measured within 24 hpi by qPCR and IFA.

### Cell Viability Assay

The cytotoxic effects of blebbistatin, mouse-PRA, human-PRA, monkey-PRA, and MYH9^1676−1791^ were evaluated using the Cell Counting Kit-8 (CCK-8) assay (Beyotime, Nanjing, China). Briefly, the cells were cultured on 96-well plates (1 × 10^5^/well) and treated with 100 μl of appropriate medium containing different concentrations of blebbistatin, mouse-PRA, human-PRA, monkey-PRA, and MYH9^1676−1791^ at 37°C for 24 h. Then the CCK-8 reagent (10 μl/well) was added and incubated for 2 h according to the manufacturer's instructions. Cell viability was measured at an absorbance of 450 nm, and the data were analyzed by GraphPad Prism.

### RNA Extraction, RT-PCR Analysis, and Quantitative RT-PCR

The total RNA was extracted from PK-15^CD163^, HEK-293T^CD163^, and BHK-21^CD163^ using TRIzol reagent (Takara, Japan) to detect the presence of the full-length CD163 gene (Table 1) by running the sample on 1% agarose gel.

The total RNA was extracted from PRRSV-infected cells with TRIzol Reagent. About 1 μg of the total RNA was converted to cDNA using PrimeScript RT Master Mix (Takara, Japan), and then amplified using FastStart Universal SYBR Green Master Mix following the manufacturer's protocol (Roche, Basle, Switzerland). Primer sequences for N protein of PRRSV and GADPH are listed in Table 2. The GADPH was used as an internal control. qPCR reactions were performed using a StepOnePlus® Real-Time PCR System (Applied Biosystems, Foster City, CA, USA), and each assay was run in triplicate. Relative quantification of target genes was calculated using the 2^−Δ*ΔCt*^ method. Absolute quantification was performed using a template consisting of recombinant plasmid pMD18-T-N containing the PRRSV-1 or PRRSV-2 ORF7 gene as previously described, to quantify the RNA copy number of the PRRSV-1 or PRRSV-2 genome (Mu et al., 2015; Li et al., 2018).

**Table 2:** Primers used to construct different species PRA and truncated domains of PRA.

**Genes**	**Forward primer (5'-3')**	**Reverse primer (5'-3')**
pCD163	GCTCTAGAATGGTGCTACTTGAAG	CGGGATCCTCATTGTACTTCAGAGTGG
mouse PRA	CGCGGATCCATGCGGGAGCTGGACGA	CCCAAGCTTCTATTCAGCTGCCTTGGCAT
human PRA	CGGGATCCATGCGCGAGCTGGATGA	CCCAAGCTTTTATTCGGCAGGTTTGGC
monkey PRA	CGCGGATCCATGCGCGAGCTGGATG	CCCAAGCTTTTATTCGGCAGGTTTGG
MYH9 (1651-1960)	ACGCGTCGACATGCGGGAGCTGGAG	CGGGATCCTCATTGTACTTCAGAGT
MYH9 (1716-1960)	CGGGATCCATGGGGGCGCTGGCGTTG	ATTCTAGATTATTCGGCAGGTTT
MYH9 (1761-1960)	ATGGATCCATGCAGATCAATACCGAC	ATTCTAGATTATTCGGCAGGTTT
MYH9 (1786-1960)	AAGGATCCATGCAGAACAAGGAGCTC	ATTCTAGATTATTCGGCAGGTTT
MYH9 (1792-1960)	ATGGATCCATGAAGCTGCAGGAGATG	ATTCTAGATTATTCGGCAGGTTT
MYH9 (1861-1960)	ATGGATCCATGTACAAGGACCAGGCG	ATTCTAGATTATTCGGCAGGTTT
MYH9 (1676-1791)	ATGTCGAC AAAAGCATGGAGGCCGAGAT	ATTCTAGTCACTTGAGCTCCTTG
MYH9 (1651-1716)	ATGTCGACATGCGGGAGCTGGAGGACAC	ATTCTAGATTTGCCGCTGCTGTTGGCAA
MYH9 (1651-1785)	ACGCGTCGACATGCGGGAGCTGGAG	ATTCTAGAGCGCTCCAGCTGTTG
MYH9 (1651-1791)	ACGCGTCGACATGCGGGAGCTGGAG	ATTCTAGTCACTTGAGCTCCTTG
MYH9^Δ1676−1791^	CCTGCAGCTTGACCAGCTTCTTCTCGTT	AGAAGAAGCTGGTCAAGCTGCAGGAG

*MYH9 (1651-1676) were synthesized by GL Biochem (Shanghai) Ltd*.

### Knockdown Assays

The siRNAs (listed in Table 2) used for targeting MYH9 were obtained from Gene Pharma Co., Ltd. The PK-15^CD163^, HEK-293T^CD163^, and BHK-21^CD163^ cells were transfected with these siRNAs according to the protocol of X-tremeGENE siRNA Transfection Reagent, respectively, and non-targeting siRNA served as a negative control. Total RNA of PK-15^CD163^, HEK-293T^CD163^, and BHK-21^CD163^ was extracted using TRIzol reagent. The transcription levels of the PRRSV N gene and MYH9 mRNA were normalized against those of GAPDH by the 2^−Δ*ΔCT*^ threshold cycle (CT) method, and the relative fold change of the PRRSV N gene and MYH9 mRNA were then calculated, respectively. The quantification was performed in StepOne Plus Real-Time PCR System (Applied Biosystems, USA). All the test samples were run in three independent experiments.

### Antibody Production

Five female BALB/c mice aged 6–8 weeks were injected subcutaneously with ~100 μg of MYH9^1676−1791^ protein mixed with complete Freund's adjuvant in a volume of 100 μl. Then, these mice were given booster doses for three times (at 14, 28, and 42 days of initial exposure) subcutaneously with a mixture containing 100 μg of MYH9^1676−1791^ protein mixed with incomplete Freund's adjuvant and the same amount of inoculum as used in the primary exposure. Finally, the serum of mice was collected and used to confirm the binding activity with MYH9^1676−1791^ protein by IFA and Western blot.

### Indirect Immunofluorescence Assay

The PK-15^CD163^, HEK-293T^CD163^, and BHK-21^CD163^ cells were seeded on coverslips in 12-well tissue culture plates and infected with SD16 or VR-2332 (1 MOI). Cells were fixed with 75% cold ethanol at 36 or 48 hpi. Meanwhile, MARC-145 and PAM cells were plated into 12-well tissue culture plates with coverslips, respectively, and infected with SD16 (0.1 MOI). Then the cells were fixed at 24 hpi as mentioned earlier and blocked with 1% bovine serum albumin (BSA) in PBS. Cells were incubated with the primary antibody (anti-PRRSV N monoclonal antibody, 6D10) and later with secondary antibodies (rhodamine-conjugated goat anti-mouse IgG antibody) (Jackson, West Grove, USA), and were washed three times as described earlier. Finally, the cells were counterstained with DAPI, and cell staining was visualized using Leica microsystems (Leica AF6000, Germany).

### Western Blot Analysis

The cells were collected and lysed with NP40 lysis buffer (Beyotime, China), and the protein concentrations were determined using a BCA protein assay kit (Thermo, USA). Western blot was performed as described previously (Mu et al., 2015). The membranes were blocked with 5% BSA and then incubated with indicated primary antibodies overnight at 4°C, followed by incubation with HRP-conjugated goat anti-mouse IgG (Jackson Laboratories, West Grove, PA, USA), which act as secondary antibodies. β-actin served as a control. Finally, the proteins were visualized using enhanced chemiluminescence (ECL) reagents (Amersham Biosciences).

### Statistical Analysis

Statistical analysis was performed using GraphPad Prism version 5.0 (GraphPad Software, San Diego, CA, USA). Differences among the groups were analyzed by one-way ANOVA followed by Bonferroni *post-hoc* test or unpaired Student's *t*-tests. Statistically significant and very significant results were defined as *P* < 0.05 and *P* < 0.01.

## Results

### Generation and PRRSV Susceptibility of PK-15, HEK-293T, and BHK-21 Cells Expressing Porcine CD163

**Figure 1:**
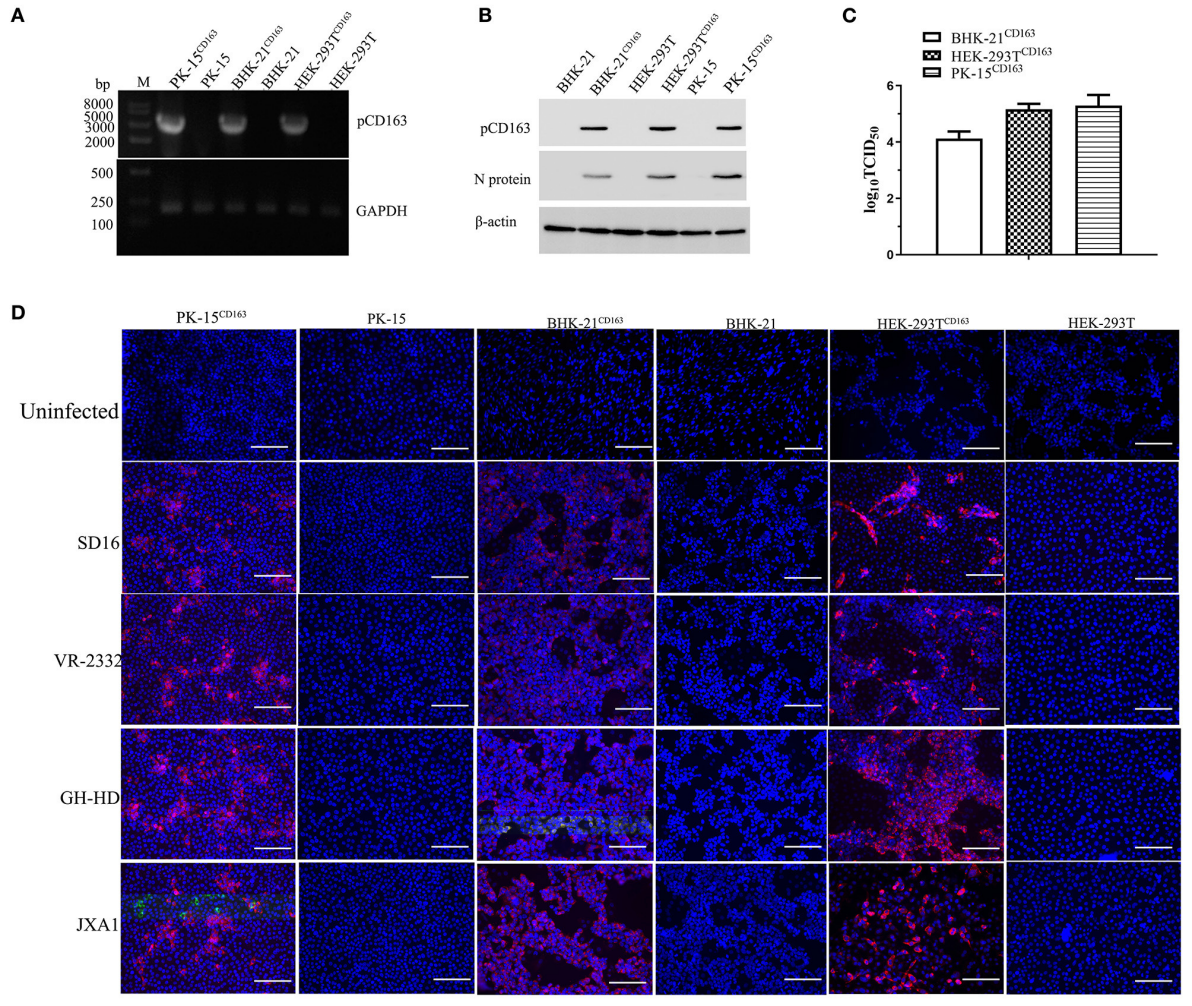
Generation of HEK-293T^CD163^, BHK-21^CD163^, and PK-15^CD163^cell lines and PRRSV infection. HEK-293T, BHK-21, and PK-15 cells were transduced with the indicated lentiviral constructs, and then these cells were selected and subcloned with puromycin. Each cell line was harvested to assess pCD163 expression. **(A)** Total RNA of each cell line was extracted and reverse transcribed to amplify the full-length gene coding region for pCD163. GAPDH transcripts were amplified to normalize the total amount of input RNA. **(B)** The expression levels of pCD163 and PRRSV N proteins were confirmed by Western blot in these cell lines infected with PRRSV SD16 at 48 hpi. **(C)** The progeny virus particles from PK-15^CD163^, HEK-293T^CD163^, and BHK-21^CD163^ at 48 hpi were titrated with MARC-145 cells. **(D)** These recombinant cell lines were inoculated with PRRSV SD16, JXA1, GD-HD, and VR-2332 strains at 1 MOI, respectively. PRRSV-specific CPEs were detected at 48 hpi with anti-N antibody (6D10) followed by rhodamine-conjugated goat anti-mouse IgG antibody, respectively. The cells were imaged using a fluorescent microscope; scale bar, 100 μm.

The HEK-293T and BHK-21 cell lines were infected with packed CD163 lentivirus according to previous studies to obtain the stable expression of pCD163 in the cell lines of human, murine, and swine species (Li et al., 2017), and the positive cell lines were obtained after 4 weeks with puromycin selection. PK-15^CD163^ was generated as previously reported in our lab (Wang et al., 2013). To determine the pCD163 expression and PRRSV susceptibility, PK-15^CD163^, HEK-293T^CD163^, and BHK-21^CD163^ cells were detected using RT-PCR, Western blot, TCID_50_, and IFA assays. First, the specific RT-PCR products (3,333 bp) were detected in these recombinant cells, while no PCR products were observed in untransduced PK-15, BHK-21, and HEK-293T cells (Figure 1A). In addition, the pCD163 expression and virus replication in these cells were determined using an anti-pCD163 polyclonal antibody and anti-PRRSV N monoclonal antibody (6D10) by Western blot, and the results suggested that pCD163 mediates PRRSV SD16 infection in PK-15, HEK-293T, and BHK-21 cells (Figure 1B). The titer values of SD16 progeny virus were determined in PK-15^CD163^, BHK-21^CD163^, and HEK-293T^CD163^ cells at 48 hpi (Figure 1C). We further determined whether stably expressing pCD163 cell lines were susceptible to PRRSV SD16, VR-2332, JXA1, and GD-HD infections using IFA. The specificity of CPE was determined using a 6D10 monoclonal antibody. The specific N protein staining was observed in PK-15^CD163^, BHK-21^CD163^ and HEK-293T^CD163^ cells at 48 hpi, while uninfected cells showed negative staining (Figure 1D).

### Decreased Basal Levels of MYH9 Reduce PRRSV Production

**Figure 2:**
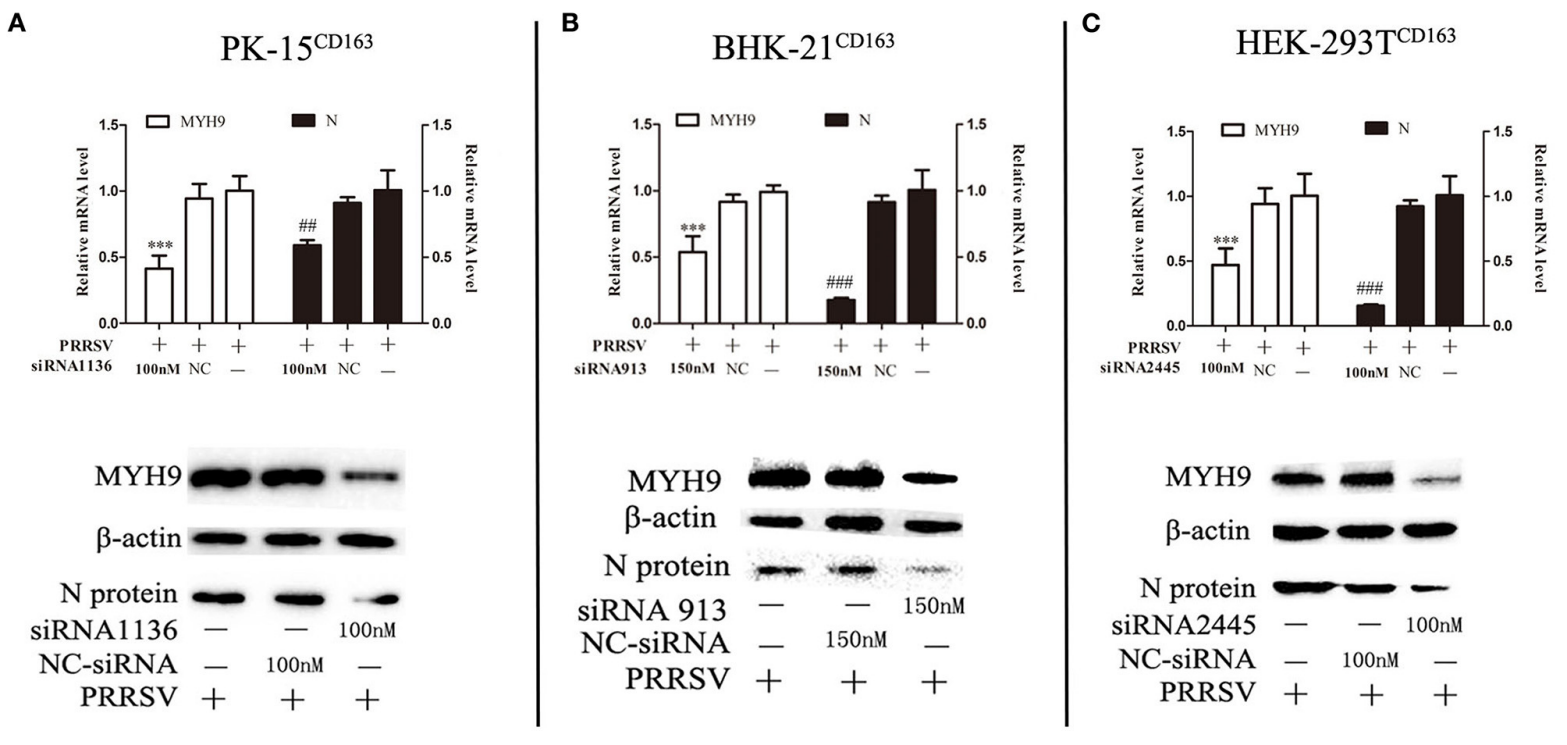
Knockdown of MYH9 of PK-15^CD163^, BHK-21^CD163^, and HEK-293T^CD163^ cell lines reduce PRRSV replication. PK-15^CD163^ **(A)**, BHK-21^CD163^ **(B)**, and HEK-293T^CD163^ **(C)** cells were transfected with MYH9 siRNA or NC siRNA, respectively, for 12 h, and then infected with PRRSV strain SD16 (1 MOI) for 48 h. PRRSV replication was measured by mRNA and protein levels using qRT-PCR and Western blot analysis. The data shown are representatives from three independent experiments and subjected to Student's *t*-test. ***P < 0.001 vs. cells treated without siRNA; ^*##*^*P* < 0.01 vs. cells treated without siRNA; ^*###*^*P* < 0.001 vs. cells treated without siRNA.

The next step was to determine whether decreased basal levels of MYH9 showed an impact on PRRSV SD16 replication in PK-15^CD163^, HEK-293T^CD163^, and BHK-21^CD163^ cells Small interfering RNA (siRNA) duplexes against MYH9 were synthesized by Gene Pharma, and non-targeting siRNA was used as control. The valid siRNA and effective concentration were determined by qPCR (Supplementary Figure 1). The cells were seeded at 60% confluence in 24-well plates before siRNA transfection. Then the cells were transfected with siRNAs for 12 h, followed by infection with SD16 at 1 MOI for 48 h. The results showed that siRNA knockdown led to a >40–50% reduction in mRNA level and protein expression of MYH9, and a significant corresponding reduction in the amount of the ORF7 mRNA and N protein expression in PRRSV SD16-infected cells compared to the negative control (Figure 2).

### Blebbistatin Inhibits PRRSV Infection

**Figure 3:**
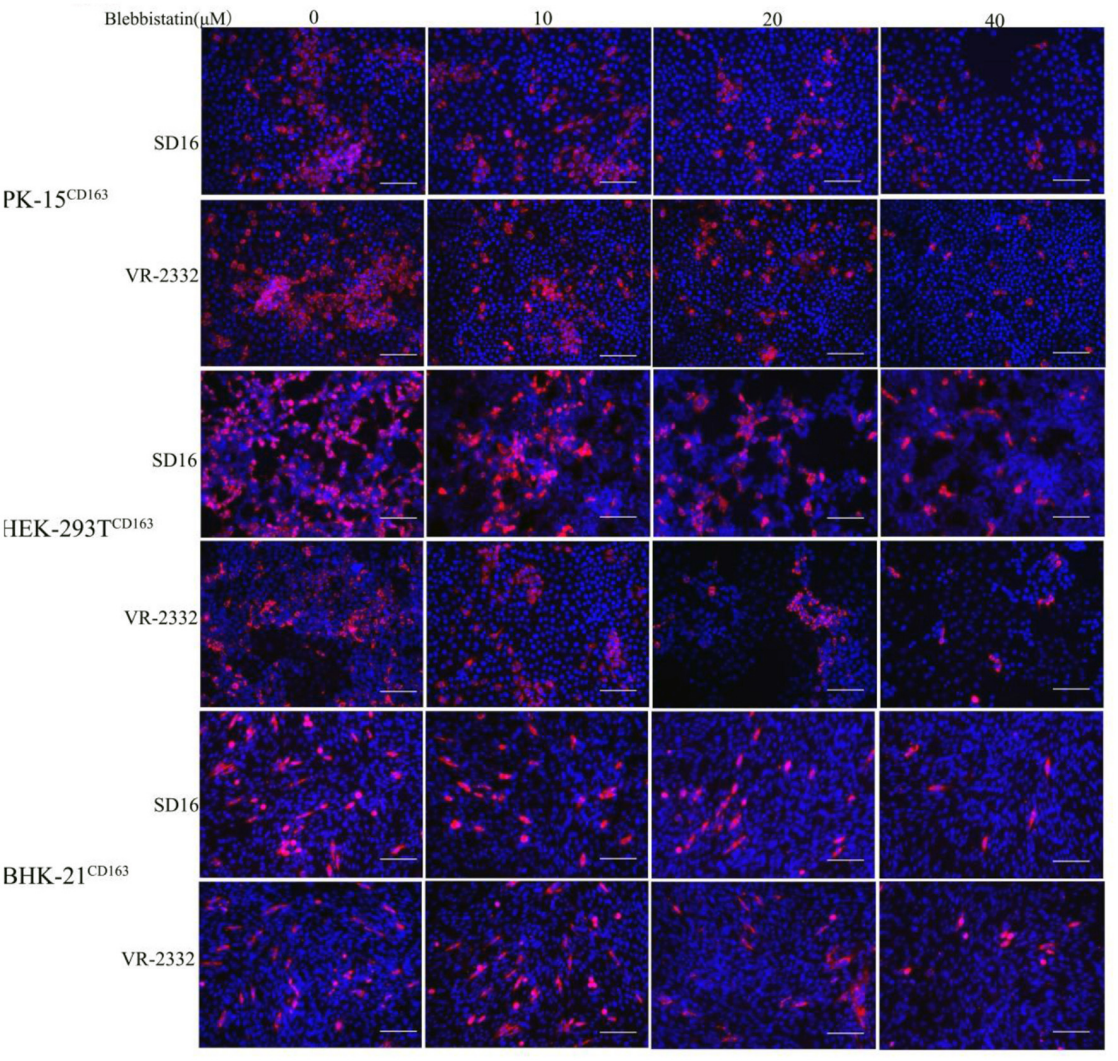
PRRSV infection in recombinant cells was inhibited by blebbistatin. PK-15^CD163^, BHK-21^CD163^, and HEK-293T^CD163^ cells were pretreated with the indicated concentrations of blebbistatin or DMSO for 30 min at 37°C, respectively, followed by incubation with PRRSV SD16 or VR-2332 at 1 MOI in the presence of blebbistatin at a concentration of 0, 10, 20, or 40 μM. These cells were fixed and stained with anti-N protein antibody (6D10) to detect PRRSV at 48 h post-infection. Images were captured using Leica microsystems (Leica AF6000, Germany). Scale bar, 100 μm.

Blebbistatin is a small molecule inhibitor of non-muscle myosin IIA (MYH9) and was shown to inhibit the MgATPase activity and *in vitro* motility of non-muscle myosin IIA (Limouze et al., 2004). We next investigated whether blebbistatin could reduce PRRSV infection in PK-15^CD163^, HEK-293T^CD163^, and BHK-21^CD163^ cell lines. These cell lines were pre-inoculated with blebbistatin and HP-PRRSV SD16 strain or classical PRRSV VR-2332 at 1 MOI, respectively, to assess the hypothesis. The specific PRRSV anti-N protein staining was performed to observe PRRSV replication at 36 hpi by IFA, and the infection efficiency was found to be significantly decreased with blebbistatin treatment (Figure 3). Therefore, the results of our study suggest that blebbistatin can block the PRRSV infection in a dose-dependent manner in these cells. Cytotoxicity assays indicated that blebbistatin exhibited little cytotoxicity against these recombinant cell lines. As expected, these results indicated that the MYH9 gene derived from human and mouse species was involved in PRRSV infection similar to the mechanism observed in the swine (Supplementary Figure 2).

### Recombinant C-Terminal end of MYH9 From Other Species Inhibits PRRSV Infection

**Figure 4:**
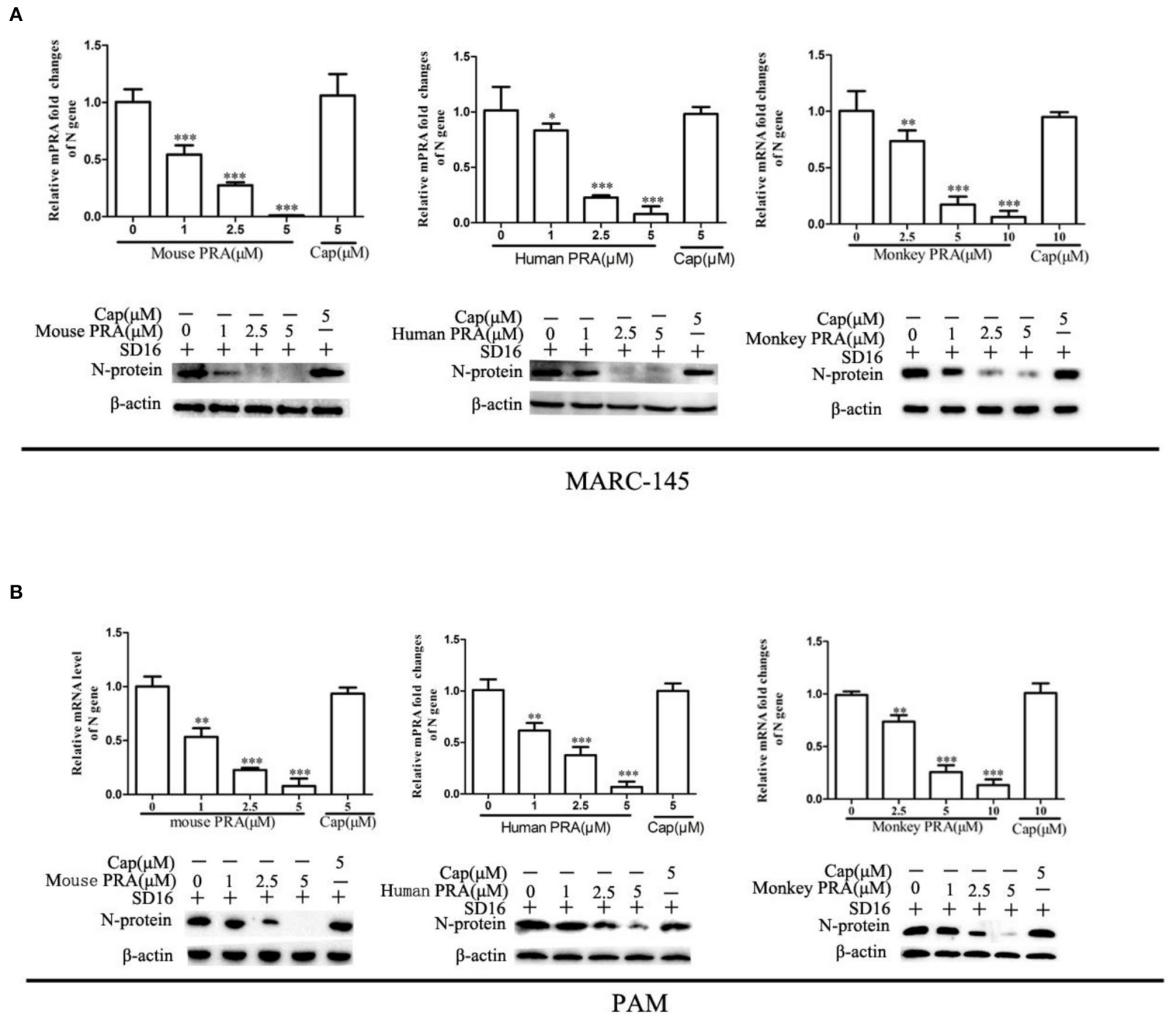
PRA from mouse, human, and monkey species inhibit PRRSV replication in the MARC-145 and PAM cells. MARC-145 cells **(A)** or PAMs **(B)** were infected with PRRSV SD16 at 0.1 MOI pre-incubated with indicated PRA protein (1, 2.5, and 5 μM), respectively, and the mRNA and protein levels of PRRSV N gene were measured at 24 hpi by qPCR and Western blot. The data shown are representatives from three independent experiments and subjected to one-way ANOVA. *P < 0.05 vs. cells treated with 0 μM PRA; **P < 0.01 vs. cells treated with 0 μM PRA; and ***P < 0.001 vs. cells treated with 0 μM PRA.

A previous study showed that the C-terminal end (aa 1651–1960) of MYH9 (PRA) from PAM cells could block the infection by different PRRSV strains via interaction with GP5 (Li et al., 2018). To assess whether mouse-PRA (mPRA) and human-PRA (hPRA) have a similar inhibitory effect in PRRSV SD16 infection as the swine PRA (sPRA) *in vitro*. MARC-145 cells served as PRRSV-susceptible cells, which were taken into consideration. The anti-PRRSV activity of the PRA domain obtained from a monkey species (monPRA) need to be tested. We obtained the recombinant PRA from different species using *E.coli* cells as previously reported (Li et al., 2018) (Supplementary Figure 3). PRRSV SD16 (0.1 MOI) was pre-incubated with indicated PRA protein of different concentrations at 37°C for 1 h, respectively, and PCV2-Cap served as control. Then the PRRSV-susceptible cells were incubated with a mixture of PRRSV and PRA proteins at 37°C for 1 h. Compared with PCV2-Cap protein treatment, PRRSV SD16 infection was blocked by pre-incubation with mPRA, hPRA, or monPRA in a dose-dependent manner, as shown by the reduction of PRRSV N protein mRNA and protein levels in MARC-145 cells (Figure 4A). The anti-PRRSV effect of indicated PRA was confirmed on PAM cells (Figure 4B), and the cytotoxicity assays indicated that PRA obtained from different species exhibited little cytotoxicity in MARC-145 and PAM cells, as expected (Supplementary Figure 4).

### Key Domain of MYH9 for PRRSV Inhibition

**Figure 5:**
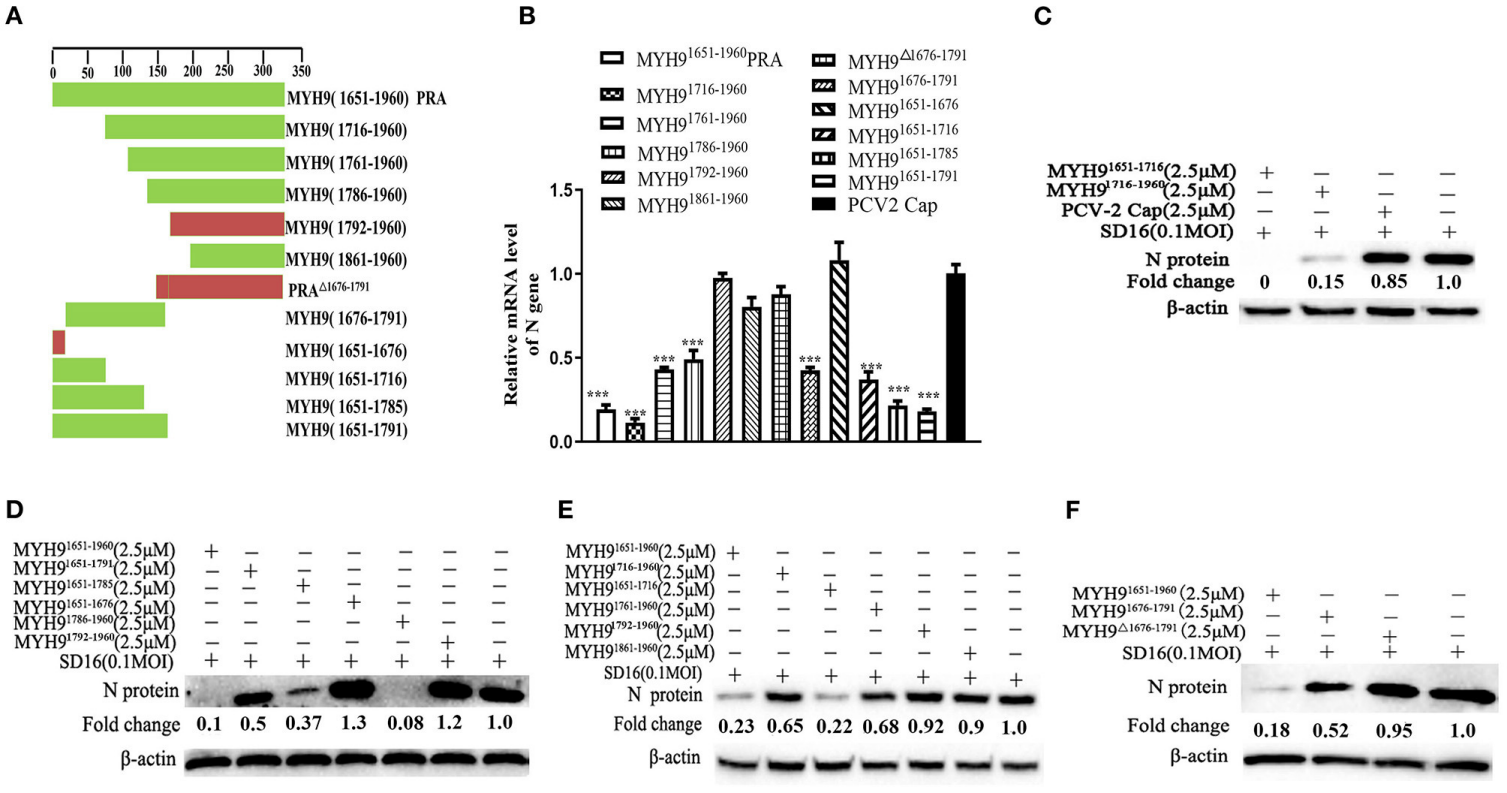
The key domain of MYH9 for PRRSV inhibition in MARC-145 cells. **(A)** Schematic diagram of truncation fragments located in the C-terminal of MYH9. MARC-145 cells were inoculated with the mixture of PRRSV 0.1 MOI and truncated protein of PRA (2.5 μM), and the key domain of MYH9 for anti-PRRSV was determined using qPCR **(B)** and Western blot **(C–F)**. The data shown are representatives from three independent experiments and subjected to one-way ANOVA. ***P < 0.001 vs. PCV2-Cap protein treated cells.

The above-mentioned results show that pre-incubation of PRRSV with PRA protein from other species could effectively inhibit PRRSV replication. Based on our previous study, the recombinant swine PRA blocks the internalization of PRRSV by direct interaction with viral GP5 (Li et al., 2018). To further identify the key region of PRA that is required for interaction with PRRSV GP5 protein, we first analyzed the sequence alignment of the amino acids constituting the PRA domain of different species using Lasergene software (Supplementary Figure 5). Based on the sequence analysis, 11 truncated fragments of swine PRA were designed (Figure 5A), and the recombinant truncated proteins without SUMO tag were obtained as previously reported (Supplementary Figure 6). Furthermore, the antiviral activity of these proteins (2.5 μM) was evaluated by pre-incubating MARC-145 cells with PRRSV SD16 (0.1 MOI) as mentioned earlier. The virus blocking assay showed that the MYH9^1676−1791^ domain could reduce PRRSV infection (Figures 5B–F), but not by the truncated fragment MYH9^Δ1676−1791^ and control PCV2 Cap protein, which suggested that the amino acid residues (1676–1791) are responsible for the interaction of MYH9 with PRRSV GP5.

**Figure 6:**
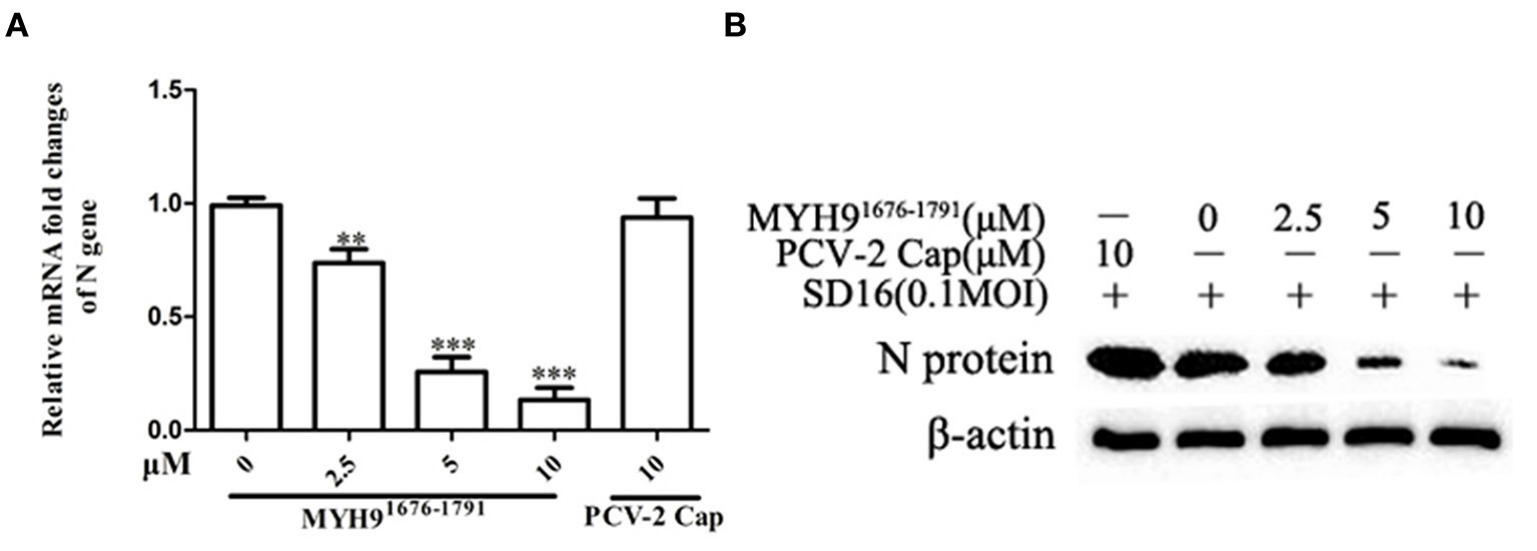
The blocking effect of MYH9^1676−1791^ against PRRSV SD16 infection in PAMs. PAMs were infected with PRRSV SD16 at 0.1 MOI pre-incubated with MYH9^1676−1791^ (2.5, 5, and 10 μM), respectively, and the mRNA **(A)** and protein levels **(B)** of PRRSV N gene were measured by qPCR and Western blot. The data shown are representatives from three independent experiments and subjected to one-way ANOVA. **P < 0.01 vs. 0 μM MYH9^1676−1791^ treated cells; ***P < 0.001 vs. 0 μM MYH9^1676−1791^ treated cells.

Porcine alveolar macrophage cells are the target cells of PRRSV infection *in vivo*. Based on the cytotoxicity assay (Supplementary Figure 7), we further evaluated the antiviral activity of MYH9^1676−1791^ protein against PAMs. As similarly inhibitor effect in MARC-145 cells. PAMs were infected with PRRSV (MOI = 0.1) pretreated with MYH9^1676−1791^ protein at various concentrations, which led to dose-dependent inhibition of viral replication at mRNA level (Figure 6A) and protein level (Figure 6B).

### Broad-Spectrum Effective Blocking of PRRSV Infection by Soluble MYH9^1676−1791^ Protein

**Figure 7:**
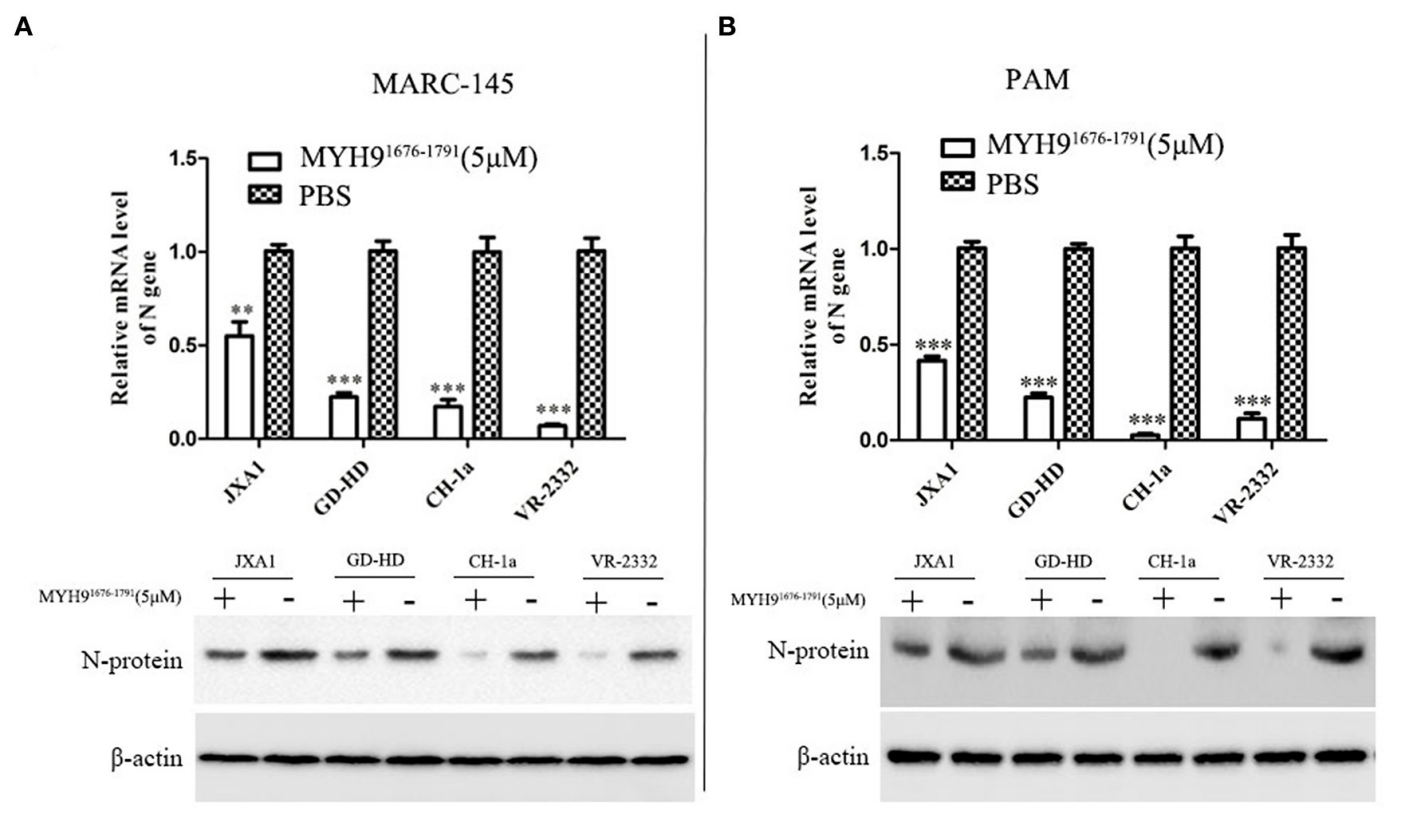
The soluble MYH9^1676−1791^ against PRRSV-2 isolates in MARC-145 and PAM cells. MARC-145 cells **(A)** or PAMs **(B)** were infected with PRRSV-2 isolates (JXA1, GD-HD, CH-1a, and VR-2332) at 0.1 MOI pre-mixed with MYH9^1676−1791^ (5 μM), and the mRNA and protein levels of N gene were measured by qPCR and Western blot. The data shown are representatives from three independent experiments and subjected to Student's *t*-test. **P < 0.01 vs. the cells infected with the same virus and treated with PBS; ***P < 0.001 vs. the cells infected with the same virus and treated with PBS.

**Figure 8:**
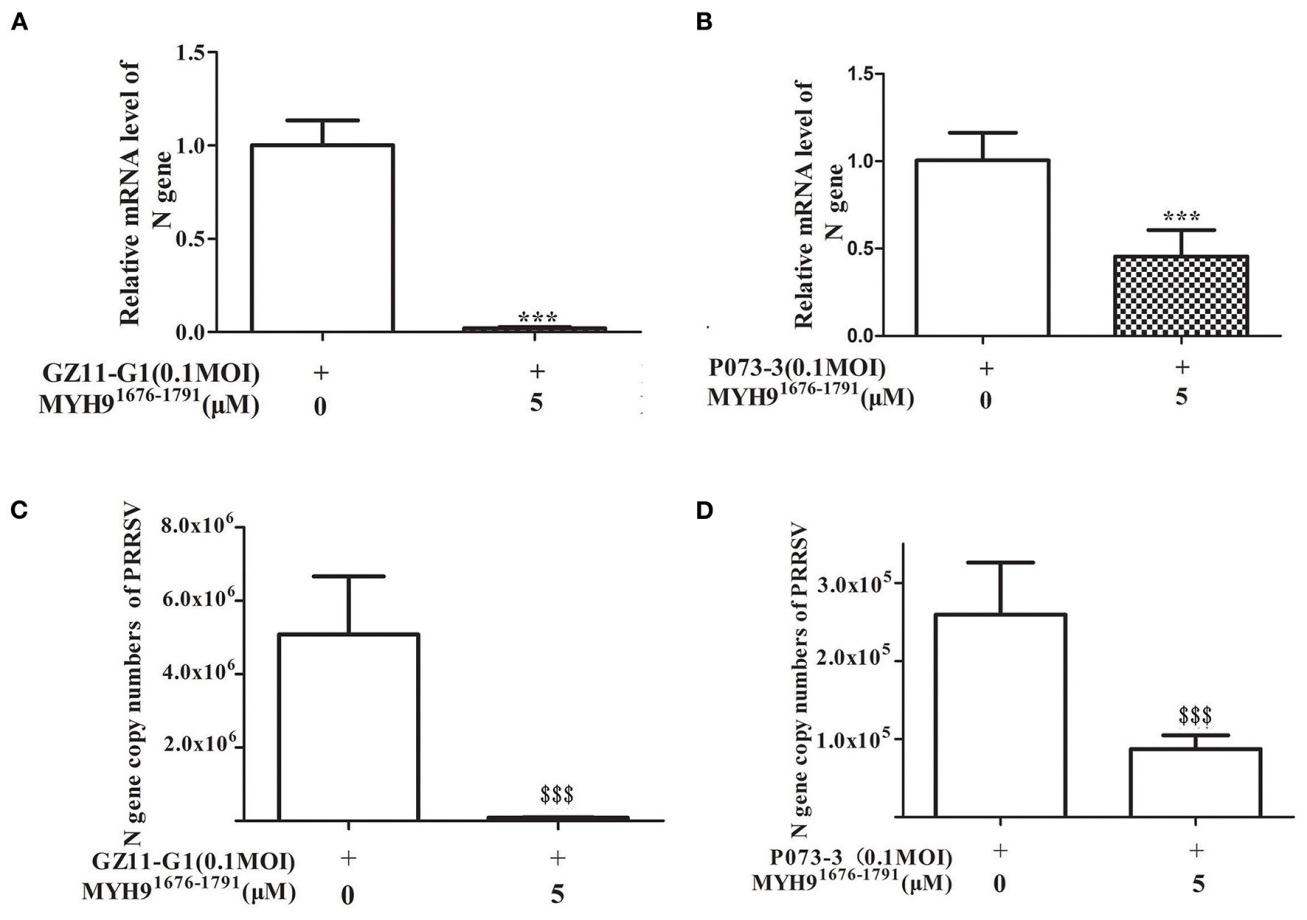
The soluble MYH9^1676−1791^ against PRRSV-1 isolates in PAM cells. MYH9^1676−1791^ (5 μM) was pre-mixed with indicated PRRSV-1 strains (MOI = 0.1) at 37°C for 1 h before infecting PAM cells. Relative PRRSV GZ11-G1 **(A)** and PRRSV P073-3 **(B)** N gene mRNA levels were monitored via qPCR at 24 hpi. The data shown are representatives from three independent experiments and subjected to Student's *t*-test. ***P < 0.001 vs. 0 μM MYH9^1676−1791^ treated cells. Gene copy numbers of PRRSV GZ11-G1 **(C)** and PRRSV P073-3 **(D)** N in cell culture supernatant at indicated MYH9^1676−1791^ concentrations of treatment were determined. The data shown are representatives from three independent experiments and subjected to Student's *t*-test. ^*$$$*^*P* < 0.001, in comparison to 0 μM MYH9^1676−1791^ treated cells.

Since the sequence alignment of aa 1676-1791 of MYH9 obtained from different species remains conservative, it could block PRRSV infection in MARC-145 and PAM cells. Next, we investigated whether the MYH9^1676−1791^ fragment could inhibit other strains of PRRSV *in vitro*. Therefore, PRRSV-2 strains CH-1a, GD-HD, JXA1, and VR-2332 were pre-incubated with 5 μM of MYH9^1676−1791^ at 37°C for 1 h as described earlier. MARC-145 or PAM cells were then infected with the mixture containing MYH9^1676−1791^ and PRRSV. Replication of these PRRSV isolates was determined based on the PRRSV N mRNA and protein levels. The result showed that the replication of PRRSV-2 strains was significantly reduced by pre-incubation with MYH9^1676−1791^ (Figures 7A,B). In addition, we tested the inhibitory effect of MYH9^1676−1791^ on PRRSV-1 isolate GZ11-G1 and P073-3, and the results showed that virus replication levels (Figures 8A,B) and ORF7 gene copies (Figures 8C,D) were significantly decreased on pre-incubation with 5 μM of MYH9^1676−1791^.

### The MYH9^1676−1791^ Protein-Specific Polyclonal Antibodies Decrease PRRSV Infection

**Figure 9:**
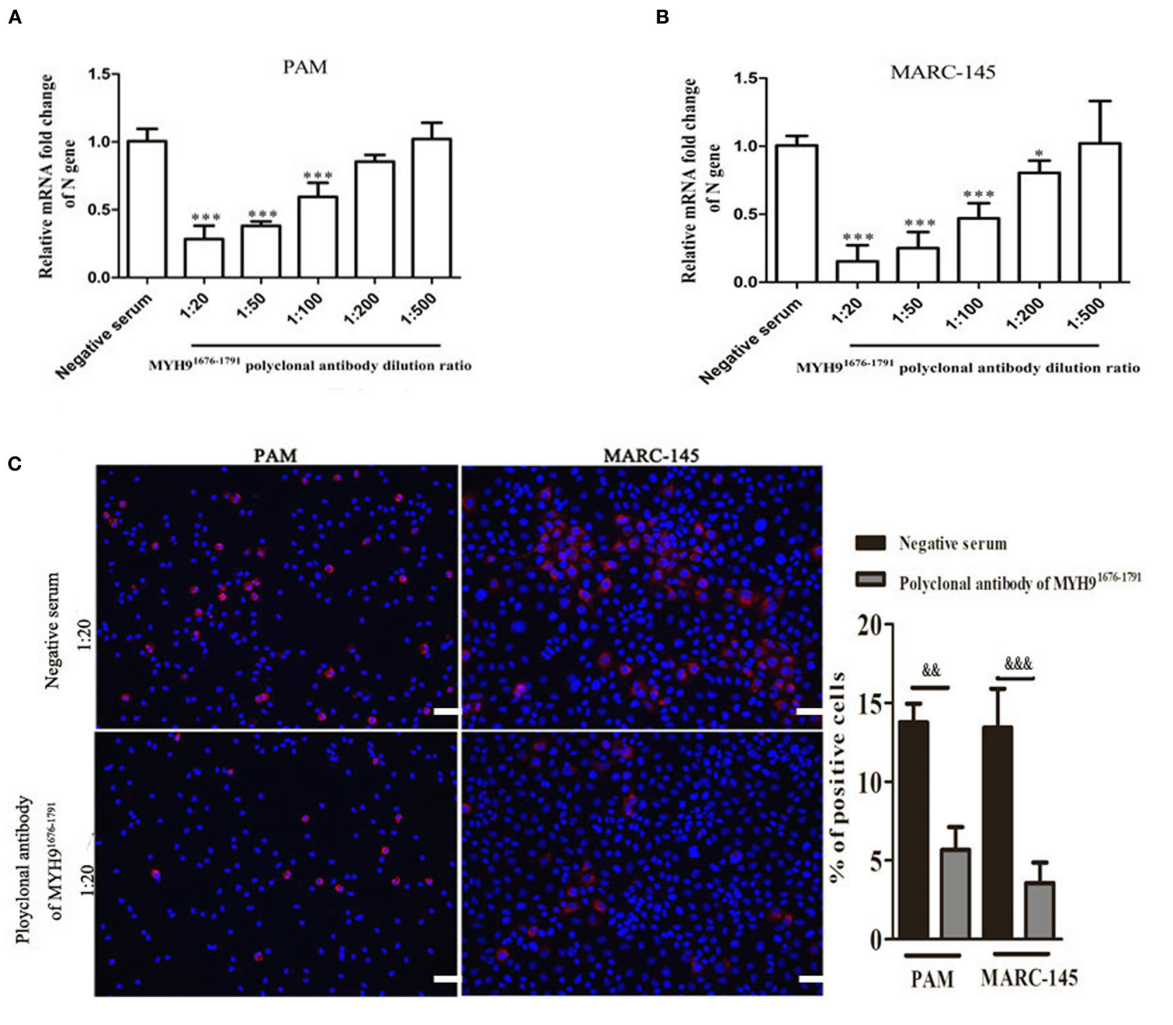
The polyclonal anti-MYH9^1676−1791^ serum reduces PRRSV infection in MARC-145 and PAM cells. MARC-145 **(A)** or PAM **(B)** cells were infected with PRRSV SD16 at an MOI of 0.1 in the presence of various concentrations of mouse anti-MYH9^1676−1791^ polyclonal serum or mouse negative serum, and relative changes in the mRNA level of PRRSV N gene were determined by qPCR at 24 hpi. The data shown are representatives from three independent experiments and subjected to one-way ANONA. *P < 0.05 vs. cells treated with mouse negative serum, ***P < 0.001 vs. cells treated with mouse negative serum. **(C)** IFA assay results confirmed the PRRSV inhibition in MARC-145 and PAM cells with anti-MYH9^1676−1791^ polyclonal serum (1:20) treated at 24 hpi (left). Scale bar, 100 μm. Histograms represent the percentage of PRRSV-positive cells, performed using ImageJ software (right). The data shown are representatives from three independent experiments and subjected to Student's *t*-test. ^&&^*P* < 0.01, ^&*&&*^*P* < 0.001.

To test whether the anti-MYH9^1676−1791^ serum could block PRRSV infection in susceptible cells, we first proposed that the MYH9 derived from MARC-145 or PAM cells could be recognized by anti-MYH9^1676−1791^ serum (Supplementary Figure 8). PAM and MARC-145 cells for antibody blocking assays were pretreated with a specific polyclonal antibody of MYH9^1676−1791^, respectively, and then serially diluted to different concentrations in the indicated cell culture medium or with control mouse IgG for 1 h at 37°C. Cells were then infected with PRRSV without removing antibodies for 1 h at 37°C. The PRRSV infection was measured by qPCR at 24 hpi, and the result showed that the polyclonal antibody of MYH9^1676−1791^ significantly reduced PRRSV infection (Figures 9A,B). An antibody blocking assay was determined by IFA in these cells with the effective antibody concentration. The PRRSV N protein staining was significantly reduced on treatment with the polyclonal antibody of MYH9^1676−1791^ (Figure 9C). These results collectively indicate that aa 1676–1791 of MYH9 plays an important role in PRRSV entry.

## Discussion

Porcine reproductive and respiratory syndrome virus (PRRSV) shows a restricted tropism for the monocyte/macrophage cell lineage. It enters cells via receptor-mediated endocytosis (Van Gorp et al., 2010). Recently, MYH9 and CD163 were reported to play an indispensable role in the PRRSV infection of MARC-145 and PAM cells (Shi et al., 2015; Gao et al., 2016; Guo et al., 2016). In our study, MYH9 homologs derived from other mammalian species showed the PRRSV entry mediator activity. In addition, we were the first to report that the key domain of MYH9 responsible for the internalization of PRRSV was located at 1676-1791 amino acids.

Porcine reproductive and respiratory syndrome virus enters the target cells by receptor-mediated endocytosis (Nauwynck et al., 1999). It is difficult for only one receptor to mediate the whole process of the virus infection, although CD163 has been reported to be sufficient to convert the non-permissive cell lines to fully permissive cell lines for PRRSV infection (Calvert et al., 2007). However, non-permissive cell lines expressing only CD163 could not be infected with PRRSV (such as COS-7, primary mouse peritoneal macrophages, and differentiated human U937 cells), as described in the previous study (Gao et al., 2016). Hence, the combined effects of these receptors are critical during the virus infection. Both CD163 and MYH9 cofactor(s) may be required for establishing a PRRSV infection (Hou et al., 2019).

Given the strict tropism of PRRSV infection, we further evaluated whether the basal levels of MYH9 derived from murine and human species showed PRRSV entry mediator activity in porcine cells. PRRSV-permissive cell lines HEK-293T^CD163^, BHK-21^CD163^, and PK-15^CD163^ were established in our study. These cell lines were transiently transfected with the siRNA targetting MYH9 or pretreated with an inhibitor (blebbistatin) to reduce MYH9 activity, and the results showed a significant decrease in PRRSV infection (Figures 2, 3). We also reported that MYH9 plays an important role during PRRSV infection of MARC-145 (monkey species) cells. These findings suggested that MYH9 may serve as a cofactor for PRRSV entry. Recently, it has been reported that the C-terminal end of MYH9 from PAM cells interacts with PRRSV GP5, thus blocking PRRSV infection (Li et al., 2018). In the present study, the soluble PRA protein from other mammalian species (human, mouse, and monkey) was obtained, and its anti-PRRSV activity was assessed (Figure 4) similar to swine PRA, which suggests that MYH9 genes derived from different species, which act as the main PRRSV entry mediators, do not account for the strict host species specificity displayed by PRRSV.

To further identify the binding domain of MYH9 from different species for PRRSV entry, we first performed the sequence alignment of the C-terminal sequence of MYH9 obtained from different mammalian species using Lasergene software (Supplementary Figure 5). We further identified the key domain (aa 1676-1791) responsible for PRRSV binding via blocking assay (Figures 5, 6). The soluble MYH9^1676−1791^ shows a broad-spectrum neutralization effect against different PRRSV strains (Figures 7, 8). Moreover, the polyclonal antibody of MYH9^1676−1791^ could reduce PRRSV infection by “occupied effect”.

In conclusion, our study demonstrated that MYH9, along with CD163, is a key cofactor that facilitates the entry of PRRSV into the host. Besides sMYH9, mMYH9 and hMYH9 also show PRRSV entry mediator activity. To date, this is the first study to establish that homologs of MYH9 can function as effective PRRSV entry mediators. Furthermore, we determined the key domain of MYH9 responsible for PRRSV internalization at 1676–1791 aa residues. Most importantly, the soluble MYH9^1676−1791^ may be useful for developing a novel agent for PRRS control and management.

## Data Availability Statement

The original contributions presented in the study are included in the article/Supplementary Material, further inquiries can be directed to the corresponding author/s.

## Author Contributions

LL performed the research, analyzed the data, and drafted the manuscript. WS, QH, and TW performed the experiment. QZ, GZ, and E-MZ revised the manuscript. All authors contributed to the revising of the manuscript.

## Funding

This work was supported by grants from the National Natural Science Foundation of China (32002248) and the Natural Science Foundation of Shandong Province (ZR2020QC016, ZR2020QC017).

## Conflict of Interest

The authors declare that the research was conducted in the absence of any commercial or financial relationships that could be construed as a potential conflict of interest.

## Publisher's Note

All claims expressed in this article are solely those of the authors and do not necessarily represent those of their affiliated organizations, or those of the publisher, the editors and the reviewers. Any product that may be evaluated in this article, or claim that may be made by its manufacturer, is not guaranteed or endorsed by the publisher.

## Supplementary Material

Supplementary Figure 1 The positive siRNAs and availability concentration targeting different species MYH9 were assessed in PK-15^CD163^, BHK-21^CD163^and HEK-293T^CD163^ cells by qPCR.

Supplementary Figure 2 The cytotoxicity assay of Blebbistatin in PK-15^CD163^, BHK-21^CD163^ and HEK-293T^CD163^ cell lines. PK-15^CD163^, BHK-21^CD163^ and HEK-293T^CD163^ cell lines were treated by increasing doses (0, 5, 10, 20, 40, and 80 μM) of Blebbistatin for 24 h and tested for viability via CCK-8 assay.

Supplementary Figure 3 Detection of PRA protein expression. PRA protein from different species (human, mouse and monkey) were expressed and purified from *E. coli* cell lysate using a His Trap HP column. The SUMO tag was removed by incubation with rTEV protease.

Supplementary Figure 4 Cytotoxicity assay of different species PRA-treated MARC-145 or PAM cell lines. MARC-145 or PAM cells were treated with increasing doses (0, 0.5, 1, 2.5, 5, and 10 μM) of different species PRA for 24 h and tested for viability via CCK-8 assay.

Supplementary Figure 5 Alignment of the PRA domain of swine MYH9, mouse MYH9, monkey MYH9 and human MYH9, dots represent residues identical to same between these species.

Supplementary Figure 6 The detection of PRA truncations expression without SUMO tag by SDS-PAGE gel analysis.

Supplementary Figure 7 Potential cytotoxicity of MYH9^1676−1791^ protein for PAM cells.. PAM cells were treated with increasing doses (0, 1, 2.5, 5, 10, and 20 μM) of MYH9^1676−1791^ for 24 h and detected with CCK-8 kit.

Supplementary Figure 8 The specificity of mouse polyclonal antibody for MYH9^1676−1791^ protein. The MYH9^1676−1791^ proteins were recognized by polyclonal antibody of MYH9^1676−1791^ using westernblot **(A)** and ELISA **(B)**.

Supplementary Figure 9 IFA assay analysis. MYH9 were recognized by anti-MYH9^aa1676−1791^ serum using IFA at the cell surface of MARC-145 or PAM during PRRSV entry. Scale bar, 100 μm.

## References

[B1] Arii J. Goto H. Suenaga T. Oyama M. Kozuka-Hata H. Imai T.. (2010). Non-muscle myosin IIA is a functional entry receptor for herpes simplex virus-1. Nature 467, 859–862. 10.1038/nature09420 20944748

[B2] Burkard C. Lillico S. G. Reid E. Jackson B. Mileham A. J. Ait-Ali T.. (2017). Precision engineering for PRRSV resistance in pigs: Macrophages from genome edited pigs lacking CD163 SRCR5 domain are fully resistant to both PRRSV genotypes while maintaining biological function. PLoS Pathog. 13, e1006206. 10.1371/journal.ppat.1006206 28231264 PMC5322883

[B3] Calvert J. G. Slade D. E. Shields S. L. Jolie R. Mannan R. M. Ankenbauer R. G.. (2007). CD163 expression confers susceptibility to porcine reproductive and respiratory syndrome viruses. J. Virol. 81, 7371–7379. 10.1128/JVI.00513-07 17494075 PMC1933360

[B4] Chen J. Fan J. Chen Z. Zhang M. Peng H. Liu J.. (2021). Nonmuscle myosin heavy chain IIA facilitates SARS-CoV-2 infection in human pulmonary cells. Proc. Natl. Acad. Sci. U. S. A. 118, e2111011118. 10.1073/pnas.2111011118 34873039 PMC8685683

[B5] Das P. B. Dinh P. X. Ansari I. H. de Lima M. Osorio F. A. Pattnaik A. K. (2010). The minor envelope glycoproteins GP2a and GP4 of porcine reproductive and respiratory syndrome virus interact with the receptor CD163. J. Virol. 84, 1731–1740. 10.1128/JVI.01774-09 19939927 PMC2812361

[B6] Das P. B. Vu H. L. Dinh P. X. Cooney J. L. Kwon B. Osorio F. A.. (2011). Glycosylation of minor envelope glycoproteins of porcine reproductive and respiratory syndrome virus in infectious virus recovery, receptor interaction, and immune response. Virology 410, 385–394. 10.1016/j.virol.2010.12.002 21195444

[B7] Delputte P. L. Vanderheijden N. Nauwynck H. J. Pensaert M. B. (2002). Involvement of the matrix protein in attachment of porcine reproductive and respiratory syndrome virus to a heparinlike receptor on porcine alveolar macrophages. J. Virol. 76, 4312–4320. 10.1128/JVI.76.9.4312-4320.2002 11932397 PMC155060

[B8] Du E. Tikoo S. K. (2010). Efficient replication and generation of recombinant bovine adenovirus-3 in nonbovine cotton rat lung cells expressing I-SceI endonuclease. J. Gene Med. 12, 840–847. 10.1002/jgm.1505 20963806

[B9] Duan X. Nauwynck H. J. Favoreel H. W. Pensaert M. B. (1998). Identification of a putative receptor for porcine reproductive and respiratory syndrome virus on porcine alveolar macrophages. J. Virol. 72, 4520–4523. 10.1128/JVI.72.5.4520-4523.1998 9557752 PMC109698

[B10] Gao J. Ji P. Zhang M. Wang X. Li N. Wang C.. (2014). GP5 expression in Marc-145 cells inhibits porcine reproductive and respiratory syndrome virus infection by inducing beta interferon activity. Vet. Microbiol. 174, 409–418. 10.1016/j.vetmic.2014.09.030 25457367

[B11] Gao J. Xiao S. Xiao Y. Wang X. Zhang C. Zhao Q.. (2016). MYH9 is an essential factor for porcine reproductive and respiratory syndrome virus infection. Sci. Rep. 6, 25120. 10.1038/srep25120 27112594 PMC4845007

[B12] Guo R. Katz B. B. Tomich J. M. Gallagher T. Fang Y. (2016). Porcine reproductive and respiratory syndrome virus utilizes nanotubes for intercellular spread. J. Virol. 90, 5163–5175. 10.1128/JVI.00036-16 26984724 PMC4859731

[B13] Hou G. Xue B. Li L. Nan Y. Zhang L. Li K.. (2019). Direct Interaction between CD163 N-terminal domain and MYH9 C-terminal domain contributes to porcine reproductive and respiratory syndrome virus internalization by permissive cells. Front. Microbiol. 10, 1815. 10.3389/fmicb.2019.01815 31447818 PMC6691103

[B14] Kim J. K. Fahad A. M. Shanmukhappa K. Kapil S. (2006). Defining the cellular target(s) of porcine reproductive and respiratory syndrome virus blocking monoclonal antibody 7G10. J. Virol. 80, 689–696. 10.1128/JVI.80.2.689-696.2006 16378972 PMC1346842

[B15] Li L. Wu C. Hou G. Xue B. Xie S. Zhao Q.. (2017). Generation of murine macrophage-derived cell lines expressing porcine CD163 that support porcine reproductive and respiratory syndrome virus infection. BMC Biotechnol. 17, 77. 10.1186/s12896-017-0399-5 29121904 PMC5680797

[B16] Li L. Xue B. Sun W. Gu G. Hou G. Zhang L.. (2018). Recombinant MYH9 protein C-terminal domain blocks porcine reproductive and respiratory syndrome virus internalization by direct interaction with viral glycoprotein 5. Antiviral Res. 156, 10–20. 10.1016/j.antiviral.2018.06.001 29879459

[B17] Li L. Zhang L. Hu Q. Zhao L. Nan Y. Hou G.. (2019). MYH9 key amino acid residues identified by the anti-idiotypic antibody to porcine reproductive and respiratory syndrome virus glycoprotein 5 involve in the virus internalization by porcine alveolar macrophages. Viruses 12, 40. 10.3390/v12010040 31905776 PMC7019770

[B18] Limouze J. Straight A. F. Mitchison T. Sellers J. R. (2004). Specificity of blebbistatin, an inhibitor of myosin II. J. Muscle Res. Cell Motil. 25, 337–341. 10.1007/s10974-004-6060-7 15548862

[B19] Ma H. Jiang L. Qiao S. Zhi Y. Chen X. X. Yang Y.. (2017). The crystal structure of the fifth scavenger receptor cysteine-rich domain of porcine CD163 reveals an important residue involved in porcine reproductive and respiratory syndrome virus infection. J. Virol. 91, e01897-16. 10.1128/JVI.01897-16 27881657 PMC5244331

[B20] Mu Y. Li L. Zhang B. Huang B. Gao J. Wang X.. (2015). Glycoprotein 5 of porcine reproductive and respiratory syndrome virus strain SD16 inhibits viral replication and causes G2/M cell cycle arrest, but does not induce cellular apoptosis in Marc-145 cells. Virology 484, 136–145. 10.1016/j.virol.2015.05.019 26093497

[B21] Nauwynck H. J. Duan X. Favoreel H. W. Van Oostveldt P. Pensaert M. B. (1999). Entry of porcine reproductive and respiratory syndrome virus into porcine alveolar macrophages via receptor-mediated endocytosis. J. Gen. Virol. 80 (Pt 2), 297–305. 10.1099/0022-1317-80-2-297 10073688

[B22] Pineyro P. E. Subramaniam S. Kenney S. P. Heffron C. L. Gimenez-Lirola L. G. Meng X. J. (2016). Modulation of proinflammatory cytokines in monocyte-derived dendritic cells by porcine reproductive and respiratory syndrome virus through interaction with the porcine intercellular-adhesion-molecule-3-grabbing nonintegrin. Viral Immunol. 29, 546–556. 10.1089/vim.2016.0104 27643915

[B23] Shi C. Liu Y. Ding Y. Zhang Y. Zhang J. (2015). PRRSV receptors and their roles in virus infection. Arch Microbiol. 197, 503–512. 10.1007/s00203-015-1088-1 25666932

[B24] Sun Y. Qi Y. Liu C. Gao W. Chen P. Fu L.. (2014). Nonmuscle myosin heavy chain IIA is a critical factor contributing to the efficiency of early infection of severe fever with thrombocytopenia syndrome virus. J. Virol. 88, 237–248. 10.1128/JVI.02141-13 24155382 PMC3911693

[B25] Teifke J. P. Dauber M. Fichtner D. Lenk M. Polster U. Weiland E.. (2001). Detection of European porcine reproductive and respiratory syndrome virus in porcine alveolar macrophages by two-colour immunofluorescence and in-situ hybridization-immunohistochemistry double labelling. J. Comp. Pathol. 124, 238–245. 10.1053/jcpa.2000.0458 11437499

[B26] Van Gorp H. Van Breedam W. Van Doorsselaere J. Delputte P. L. Nauwynck H. J. (2010). Identification of the CD163 protein domains involved in infection of the porcine reproductive and respiratory syndrome virus. J. Virol. 84, 3101–3105. 10.1128/JVI.02093-09 20032174 PMC2826032

[B27] Vicente-Manzanares M. Ma X. Adelstein R. S. Horwitz A. R. (2009). Non-muscle myosin II takes centre stage in cell adhesion and migration. Nat. Rev. Mol. Cell Biol. 10, 778–790. 10.1038/nrm2786 19851336 PMC2834236

[B28] Wang X. Wei R. Li Q. Liu H. Huang B. Gao J.. (2013). PK-15 cells transfected with porcine CD163 by PiggyBac transposon system are susceptible to porcine reproductive and respiratory syndrome virus. J. Virol Methods 193, 383–390. 10.1016/j.jviromet.2013.06.035 23835031

[B29] Wells K. D. Bardot R. Whitworth K. M. Trible B. R. Fang Y. Mileham A.. (2017). Replacement of porcine CD163 scavenger receptor cysteine-rich domain 5 with a CD163-like homolog confers resistance of pigs to genotype 1 but not genotype 2 porcine reproductive and respiratory syndrome virus. J. Virol. 91, e01521-16. 10.1128/JVI.01521-16 27847356 PMC5215333

[B30] Wu J. Peng X. Zhou A. Qiao M. Wu H. Xiao H.. (2014). MiR-506 inhibits PRRSV replication in MARC-145 cells via CD151. Mol. Cell Biochem. 394, 275–281. 10.1007/s11010-014-2103-6 24878990

[B31] Xiong D. Du Y. Wang H. B. Zhao B. Zhang H. Li Y.. (2015). Nonmuscle myosin heavy chain IIA mediates Epstein-Barr virus infection of nasopharyngeal epithelial cells. Proc. Natl. Acad. Sci. U. S. A. 112, 11036–11041. 10.1073/pnas.1513359112 26290577 PMC4568263

[B32] Xue B. Hou G. Zhang G. Huang J. Li L. Nan Y.. (2019). MYH9 aggregation induced by direct interaction with PRRSV GP5 ectodomain facilitates viral internalization by permissive cells. Front. Microbiol. 10, 2313. 10.3389/fmicb.2019.02313 31649651 PMC6794372

