# Supplementary figures and images for: Identification of MYH9 Key Domain Involved in the Entry of PRRSV Into Permissive Cells
Source: Front Microbiol. 2022 May 25;13:865343. doi: 10.3389/fmicb.2022.865343 (PMC9174932; doi:10.3389/fmicb.2022.865343)

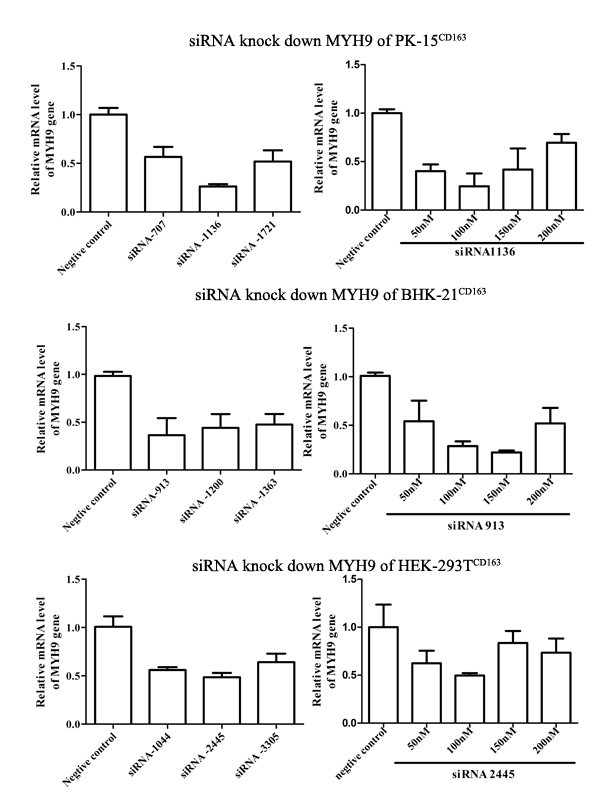

Supplement: Supplementary Figure 1 — The positive siRNAs and availability concentration targeting different species MYH9 were assessed in PK-15CD163, BHK-21CD163and HEK-293TCD163 cells by qPCR. [file Image_1.tif]

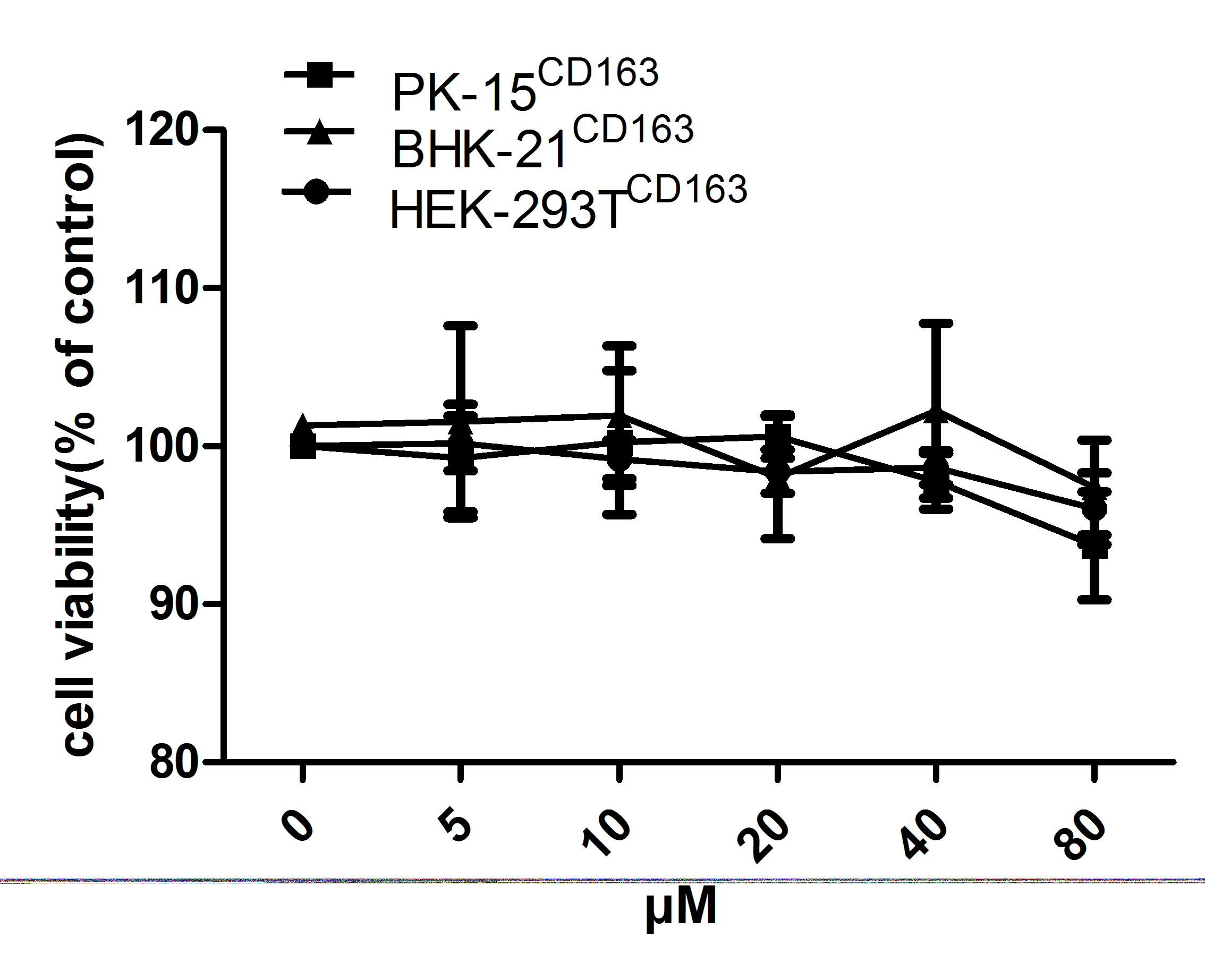

Supplement: Supplementary Figure 2 — The cytotoxicity assay of Blebbistatin in PK-15CD163, BHK-21CD163 and HEK-293TCD163 cell lines. PK-15CD163, BHK-21CD163 and HEK-293TCD163 cell lines were treated by increasing doses (0, 5, 10, 20, 40, and 80 μM) of Blebbistatin for 24 h and tested for viability via CCK-8 assay. [file Image_2.tif]

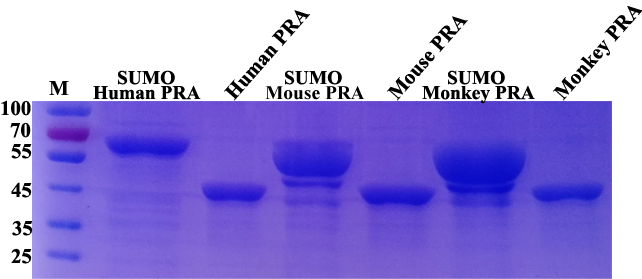

Supplement: Supplementary Figure 3 — Detection of PRA protein expression. PRA protein from different species (human, mouse and monkey) were expressed and purified from E. coli cell lysate using a His Trap HP column. The SUMO tag was removed by incubation with rTEV protease. [file Image_3.tif]

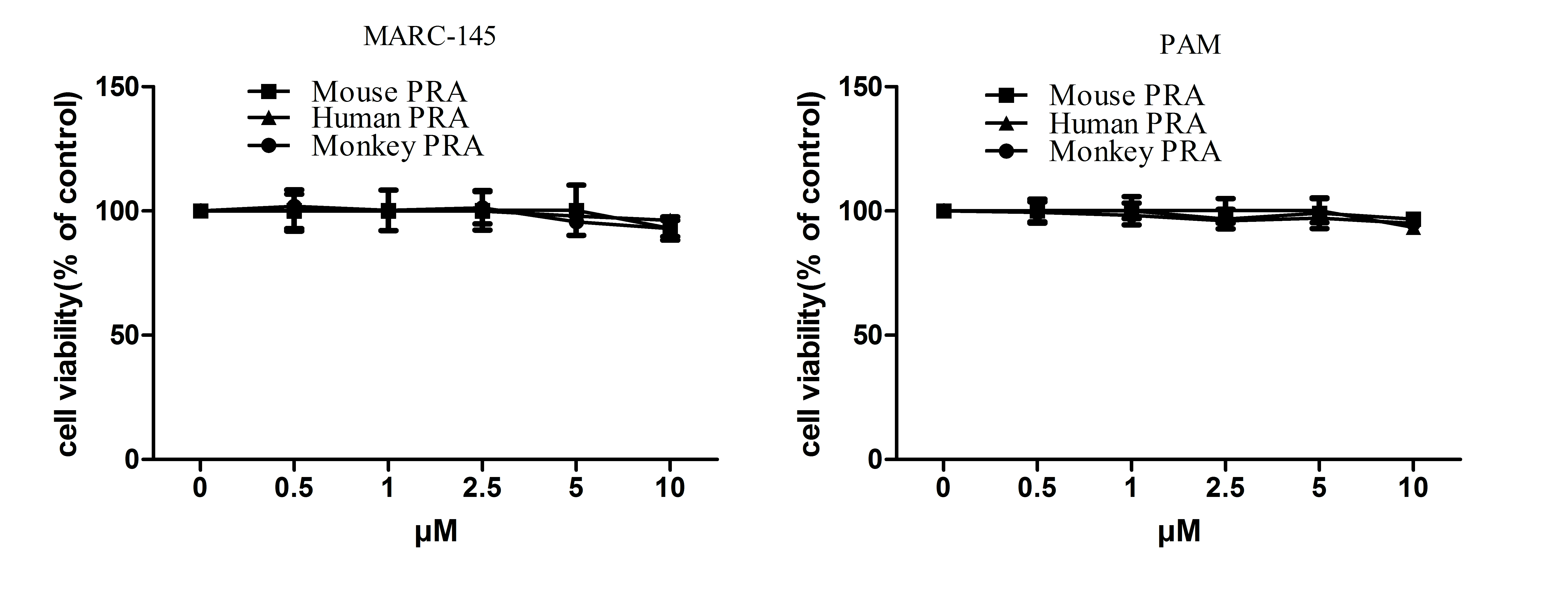

Supplement: Supplementary Figure 4 — Cytotoxicity assay of different species PRA-treated MARC-145 or PAM cell lines. MARC-145 or PAM cells were treated with increasing doses (0, 0.5, 1, 2.5, 5, and 10 μM) of different species PRA for 24 h and tested for viability via CCK-8 assay. [file Image_4.tif]

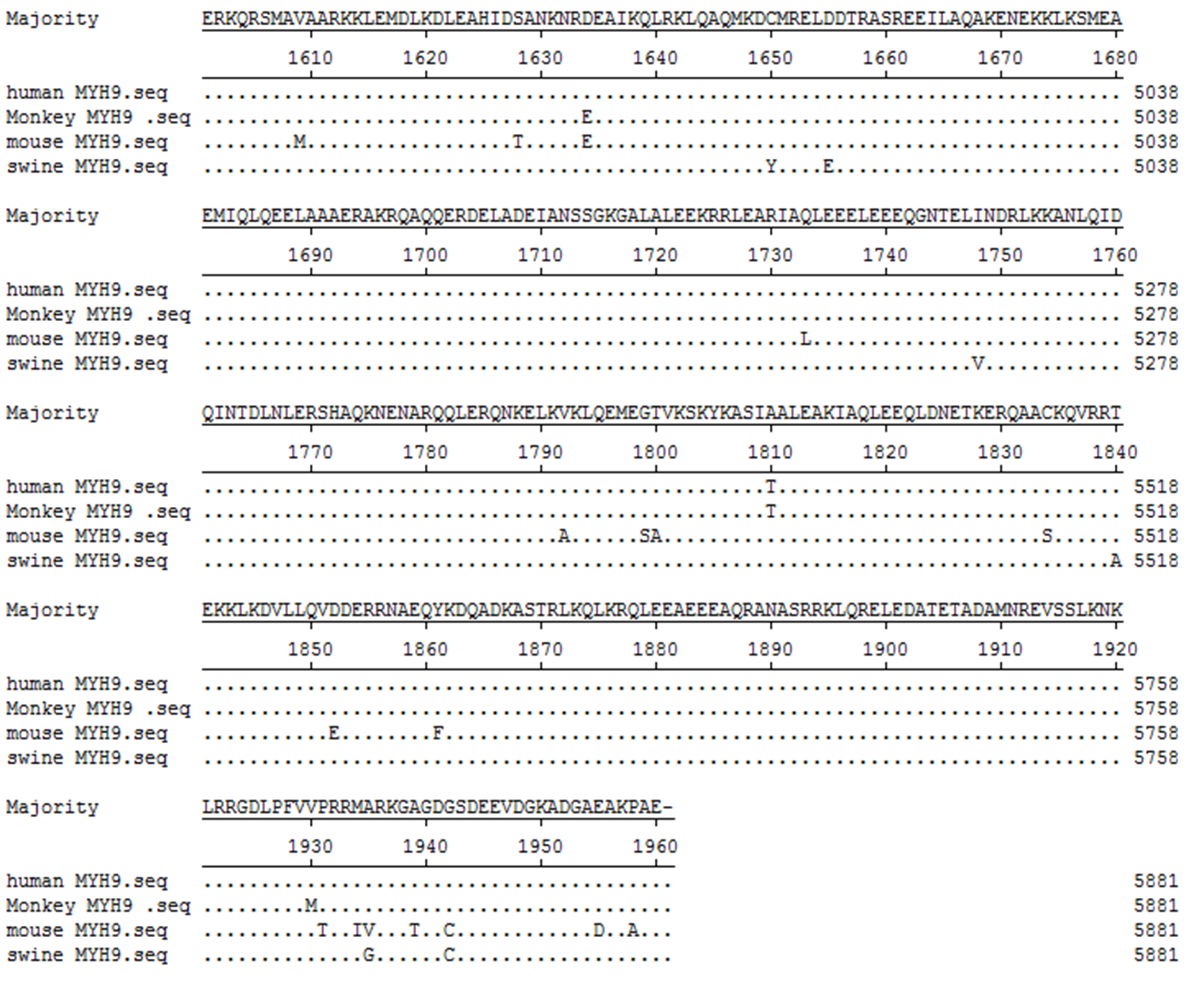

Supplement: Supplementary Figure 5 — Alignment of the PRA domain of swine MYH9, mouse MYH9, monkey MYH9 and human MYH9, dots represent residues identical to same between these species. [file Image_5.tif]

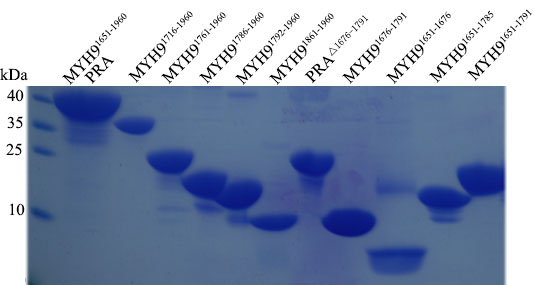

Supplement: Supplementary Figure 6 — The detection of PRA truncations expression without SUMO tag by SDS-PAGE gel analysis. [file Image_6.tif]

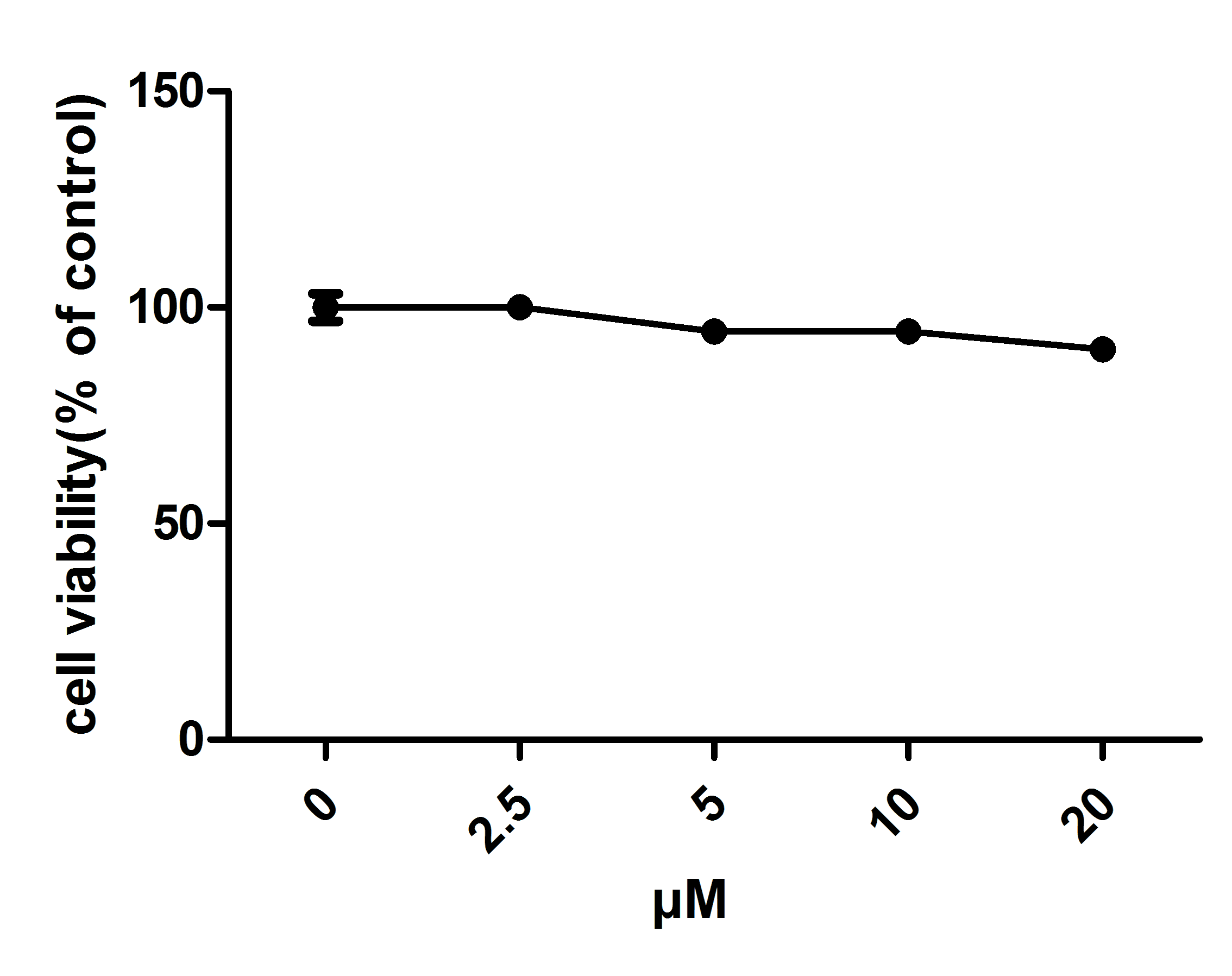

Supplement: Supplementary Figure 7 — Potential cytotoxicity of MYH91676−1791 protein for PAM cells.. PAM cells were treated with increasing doses (0, 1, 2.5, 5, 10, and 20 μM) of MYH91676−1791 for 24 h and detected with CCK-8 kit. [file Image_7.tif]

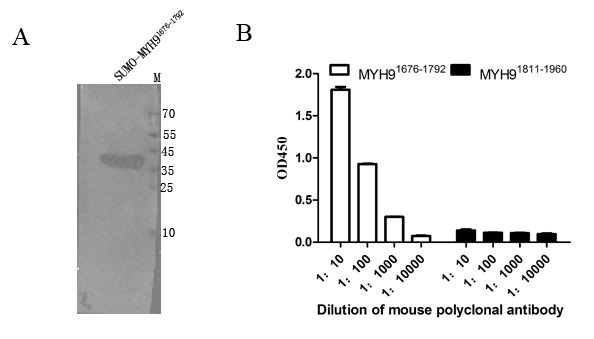

Supplement: Supplementary Figure 8 — The specificity of mouse polyclonal antibody for MYH91676−1791 protein. The MYH91676−1791 proteins were recognized by polyclonal antibody of MYH91676−1791 using westernblot (A) and ELISA (B). [file Image_8.tif]

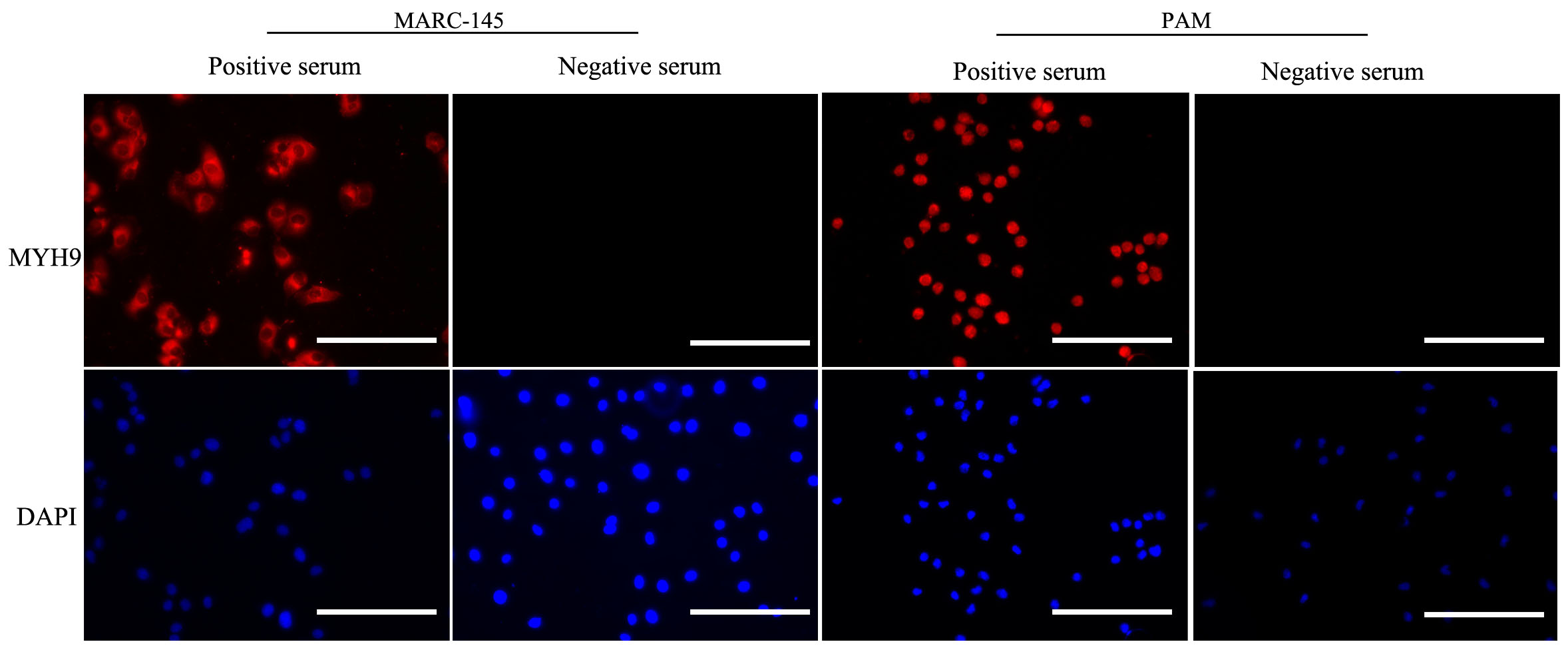

Supplement: Supplementary Figure 9 — IFA assay analysis. MYH9 were recognized by anti-MYH9aa1676−1791 serum using IFA at the cell surface of MARC-145 or PAM during PRRSV entry. Scale bar, 100 μm. [file Image_9.tif]
